# Vancomycin tolerance of adherent *Staphylococcus aureus* is impeded by nanospike-induced physiological changes

**DOI:** 10.1038/s41522-023-00458-5

**Published:** 2023-11-29

**Authors:** Andrew Hayles, Richard Bright, Ngoc Huu Nguyen, Vi Khanh Truong, Jonathan Wood, Dennis Palms, Jitraporn Vongsvivut, Dan Barker, Krasimir Vasilev

**Affiliations:** 1 College of Medicine and Public Health, Flinders University, Bedford Park, SA 5042 Australia; 2 School of Biomedical Engineering, Faculty of Engineering, University of Sydney, Sydney, Australia; 3 Academic Unit of STEM, University of South Australia, Mawson Lakes, Adelaide, 5095 SA Australia; 4 Infrared Microspectroscopy (IRM) Beamline, ANSTO ‒ Australian Synchrotron, 800 Blackburn Road, Clayton, VIC 3168 Australia; 5 Corin Australia, Baulkham Hills, NSW 2153 Australia

**Keywords:** Clinical microbiology, Antimicrobials

## Abstract

Bacterial colonization of implantable biomaterials is an ever-pervasive threat that causes devastating infections, yet continues to elude resolution. In the present study, we report how a rationally designed antibacterial surface containing sharp nanospikes can enhance the susceptibility of pathogenic bacteria to antibiotics used in prophylactic procedures. We show that *Staphylococcus aureus*, once adhered to a titanium surface, changes its cell-surface charge to increase its tolerance to vancomycin. However, if the Ti surface is modified to bear sharp nanospikes, the activity of vancomycin is rejuvenated, leading to increased bacterial cell death through synergistic activity. Analysis of differential gene expression provided evidence of a set of genes involved with the modification of cell surface charge. Synchrotron-sourced attenuated Fourier-transform infrared microspectroscopy (ATR-FTIR), together with multivariate analysis, was utilized to further elucidate the biochemical changes of *S. aureus* adhered to nanospikes. By inhibiting the ability of the pathogen to reduce its net negative charge, the nanoengineered surface renders *S. aureus* more susceptible to positively charged antimicrobials such as vancomycin. This finding highlights the opportunity to enhance the potency of prophylactic antibiotic treatments during implant placement surgery by employing devices having surfaces modified with spike-like nanostructures.

## Introduction

The rate of implant-related bacterial infections has remained stubbornly unchanged over the last decades. Such infections can be devastating to patients, causing complicated revision surgeries, amputations, and even death. Prophylactic administration of antibiotics plays a crucial role in protecting patients from infections. However, the rise of antibiotic resistance and the complex nature of bacterial colonization of implant surfaces have led to serious concerns about the long-term effectiveness of antibiotics, such as vancomycin, often used in prophylactic treatment. Bacterial colonization of an implanted material results in the formation of a biofilm on the foreign surface. Biofilm enables the pathogen to evade the host immune system^1^ and persist through antibiotic treatment^2^. It is well established that biofilms increase the drug tolerance of bacteria by as much as 1000-fold^3^. As antibiotics alone are insufficient, implant infections typically require further invasive revision surgeries. The results can be disastrous for patients, often resulting in limb amputation^4^ and even death^5^. Various biofilm-associated factors are purported to contribute to the increased antibiotic tolerance of the pathogens. Some of these include the secretion of extracellular polymeric substance (EPS)^6^, reduced metabolic activity^7^, and modification of cell surface structures^8^.

In an ideal situation, infection would be mitigated if bacteria are prevented from attaching to the medical device surface. In pursuit of this goal, researchers have drawn upon inspiration from the natural topography of dragonfly^9^ and cicada^10^ wings to fabricate surface topographies that mechanically kill adherent bacteria. This type of mechano-bactericidal surface has been developed on silicon^11^ and titanium^12^, among other substrates. While this approach and many other technologies developed over the last decades have shown much promise, very few have been implemented on medical devices due to technological or regulatory considerations. Furthermore, antibacterial surface modification may not be sufficient to prevent all device-associated infections alone and prophylactic antibiotic treatment will remain in practice for the foreseeable future. In this study, we seek to answer the question of how bacteria are affected by prophylactically applied antibiotics during the initial stages of attachment to a rationally designed bactericidal surface containing sharp nanospikes and compare them to an unmodified control surface^13^. We hypothesize that such bactericidal surfaces containing sharp nanospikes can work synergistically with antibiotics to prevent surface contamination by invading pathogens at the early stages of attachment. For this study, we chose titanium as the substrate due to the widespread use of this material for many implant applications, such as those in orthopaedics and dentistry^14^. We selected *Staphylococcus aureus* as the studied bacterium due to the prevalence of this pathogen in medical device-associated infections^15^. We used vancomycin as the evaluated antibiotic since it is often used in prophylactic treatment procedures^16^. Utilizing a combination of microscopy, differential gene expression, and ATR-FTIR we demonstrate how the physiological characteristics of *S. aureus* are influenced, and how this leads to a synergistic antibacterial effect. The findings provide strong support for the continuing effort to combat the ever-increasing antibiotic tolerance by bacteria and ultimately development of resistance. In particular, our findings show that mechano-bactericidal nanostructures have the potential to enhance the efficacy of prophylactic antibiotic treatment. This approach is potentially paradigm-shifting. This is because, although antibiotic prophylaxis is a necessary component of implant placement surgery, its success rate is still imperfect, often leading to bacterial tolerance to high concentrations of antibiotics, causing infection and implant failure. The results of our approach highlight a promising path toward improving clinical practice.

## Results

### The influence of nanospikes on adherent *S. aureus*

**Fig. 1:**
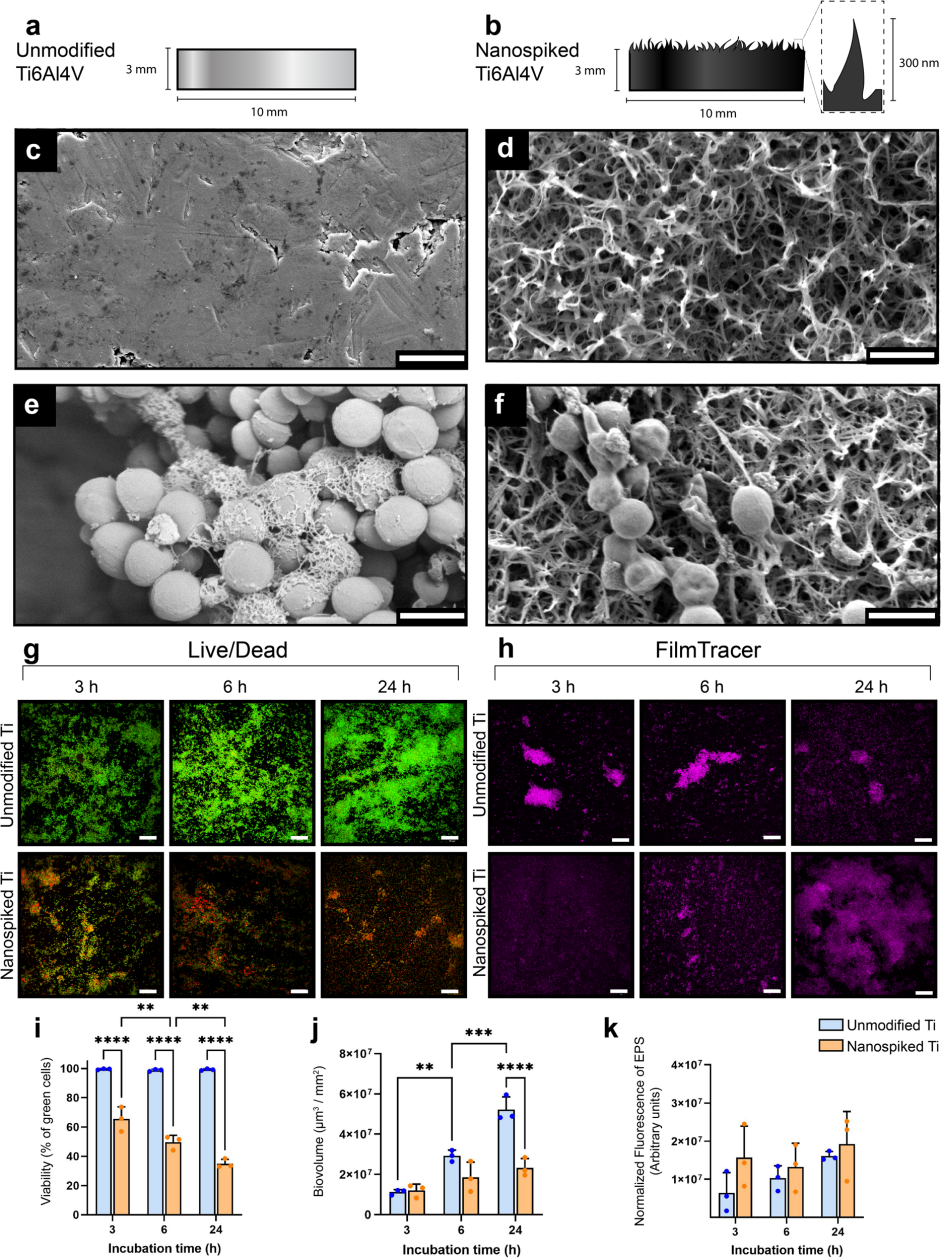
The antibacterial effect of nanospiked titanium against *S. aureus* over 24 h. **a** and **b** Schematic illustration of unmodified and nanospiked Ti samples. **c** and **d** The Ti surfaces before and after hydrothermal treatment, respectively. **e** and **f** *S. aureus* morphology following attachment to unmodified and nanospiked Ti surfaces, respectively. **g** Live/Dead fluorescence micrographs showing the accumulation of bacterial cell death on nanospiked Ti over 24 h. **h** FilmTracer™ SYPRO™ Ruby fluorescence micrographs showing the secretion of EPS over 24 h. **i** Quantified bacterial cell viability. **j** Biovolume generated from 3D z-stack fluorescence micrographs. **k** Quantification of EPS secretion using fluorescence intensity acquired from FilmTracer micrographs. **P < 0.01, ***P < 0.001 ****P < 0.0001, *n* = 3, mean ± SD. Statistical tests were performed with Two-way ANOVA using Tukey’s multiple comparisons tests. Scale bars represent 1 µm for SEM images and 30 µm for fluorescence micrographs.

We fabricated sharp nanospikes on Ti surfaces using a hydrothermal etching process^17^. Briefly, Ti6Al4V coupons (10 mm × 3 mm) were heated to 150 °C in a sealed steel vessel filled with 1 M KOH, for 5 h. SEM images show the sharp, branching and stochastically oriented features that extend from the titanium surface after modification (Fig. 1c), while the original surface is completely featureless containing only a few cracks and defects (Fig. 1d). Comprehensive characterization of the morphological, chemical and physical properties of the nanospiked and unmodified Ti samples are reported in Supplementary Fig. 1. We investigated the early stages of *S. aureus* attachment and colonization on unmodified and nanospiked surfaces using electron and fluorescence microscopy (Fig. 1e–h). After 24 h, *S. aureus* cells often appeared shrivelled, flattened or otherwise malformed on the nanospiked surface (Fig. 1f). In comparison, the cells showed a typical turgid morphology on the unmodified surface (Fig. 1e). Live/Dead staining showed that the bactericidal effect of the nanospikes was cumulative over 24 h, where approximately 35% of cells were killed after 3 h and 65% of cells were killed after 24 h. In parallel, the biovolume of *S. aureus* on the nanospiked surface did not significantly increase over 24 h, while the biovolume on the unmodified counterpart was two-fold greater by 24 h. We measured the rate of EPS secretion using the FilmTracer™ SYPRO™ Ruby Biofilm Matrix stain, which revealed approximately equal mean fluorescence intensities on both surfaces over 24 h of incubation. The FilmTracer fluorescence images revealed that EPS tended to accumulate in cell clusters on the unmodified Ti surface, while on the nanospiked Ti surface, it was more homogenously distributed across the surface.

The bactericidal efficacy of the nanospikes presented here is comparable to other reported nanostructured surfaces^18^. When Gram-positive cells are exposed to bactericidal nanostructured surfaces, most cells are killed, while a smaller subset of the population persists. This is particularly evident in Fig. 1f, where approximately half of the cells appear turgid while the other half appear severely deformed. Next, we tested our hypothesis that nanospiked surfaces induce molecular and biochemical changes in *S. aureus* which sensitize them to antibiotic treatment.

### The influence of early-stage surface attachment on the antibiotic tolerance of *S. aureus*

**Fig. 2:**
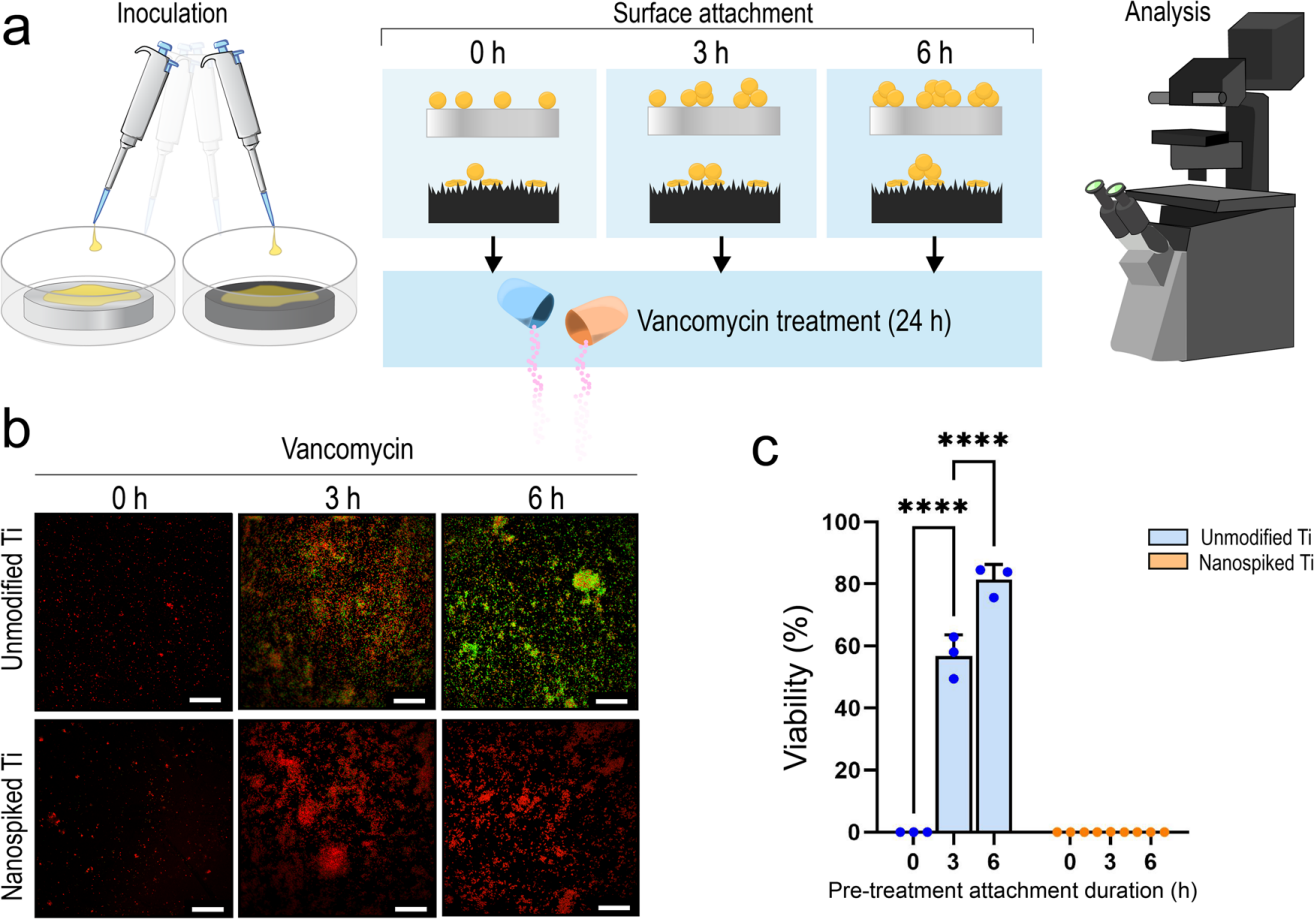
The antibiotic defences associated with surface-attached bacteria are inhibited by the nanospiked surface. **a** A flow schematic showing the attachment and treatment of *S. aureus* using vancomycin and nanospiked or unmodified surfaces. **b** Fluorescence micrographs obtained using Live/Dead staining reagent. Scale bars represent 30 µm. **c** Viability of *S. aureus* quantified from fluorescence images at 1x MIC. ****P < 0.0001, *n* = 3, mean ± SD.

It is commonly understood that bacteria are substantially more tolerant to antibiotics when they establish a biofilm^7^. In the present study, we set out to examine the change in vancomycin tolerance of *S. aureus* cells attaching to either unmodified or nanospiked Ti surfaces (Fig. 2). Vancomycin has a molecular mass of 1449.3 g/mol and a net charge of (+)0.9 at physiological pH (Supplementary Fig. 2) and is an antibiotic regularly used for prophylaxis prior to surgery. Since the goal of this study is to reveal the susceptibility of bacteria to antibiotics at early stages of surface attachment and colonization, we treated *S. aureus* with vancomycin at its minimum inhibitory concentration (MIC), for 24 h, either at the same time as inoculation or following surface attachment for 3 and 6 h. For both surfaces, when the drug was administered at the same time as inoculation, all cells were observed dead following treatment. When the cells were attached to the unmodified Ti for 3 h before vancomycin treatment, the post-treatment viability climbed to 57% and subsequently increased to 81% when the cells were attached for 6 h before vancomycin treatment. On the nanospiked Ti surface, attaching the cells to the surface for 3 and 6 h did not increase the post-treatment viability, and the entire culture was observed to be dead.

### Differential expression of genes related to formation and modification of *S. aureus* cell wall and associated structures

**Fig. 3:**
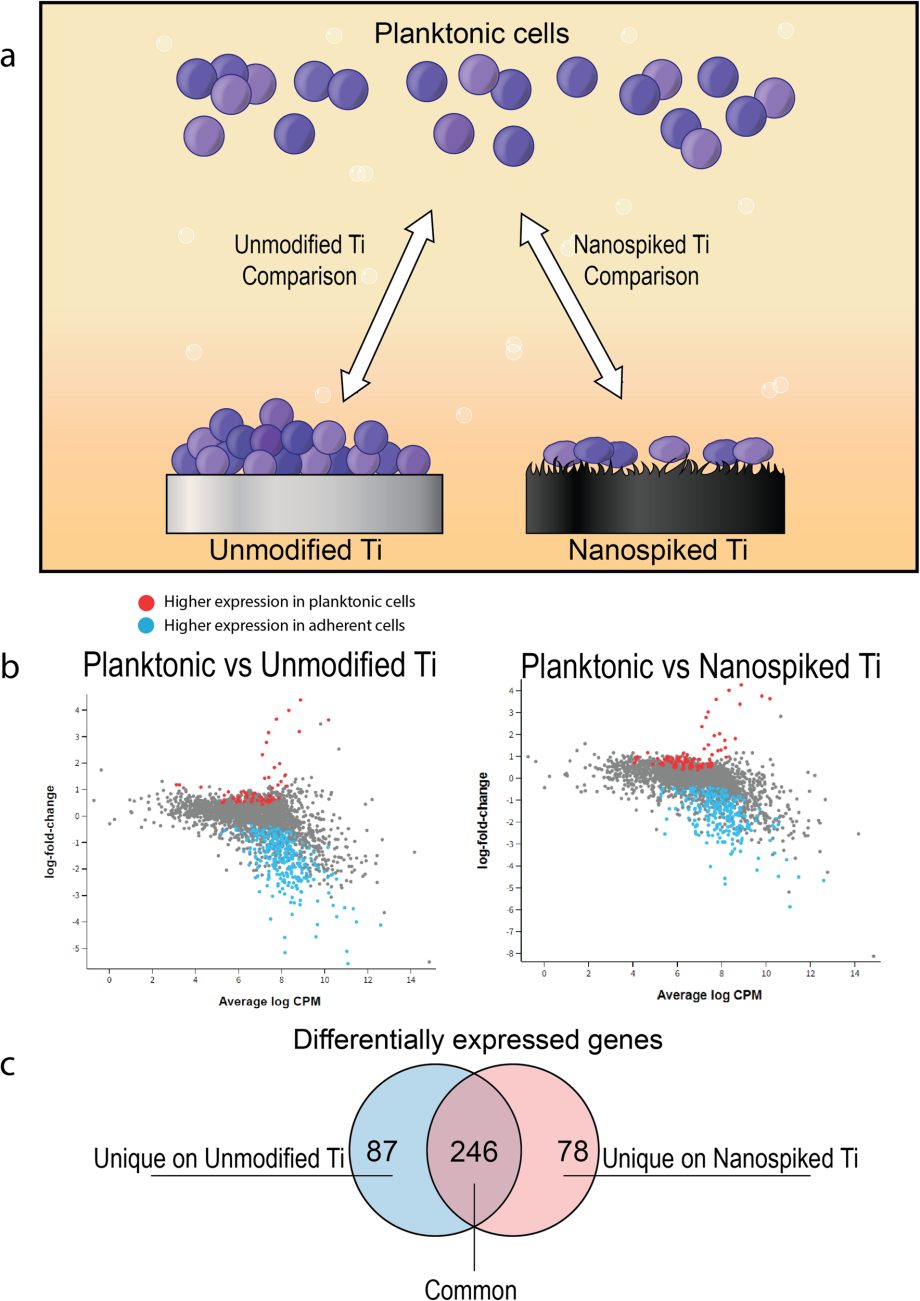
Differential gene expression analysis of planktonic and adherent *S. aureus* on nanospiked Ti. **a** A schematic of the experimental design of the differential gene expression analysis. **b** Volcano plots of differentially expressed *S. aureus* genes comparing planktonic and adherent cells on both surface types. **c** Venn diagram showing the number of common and unique differentially expressed genes from the two comparisons.

To begin to understand how adherent *S. aureus* persists through vancomycin treatment (or fails to, as on the nanospiked surface), we performed a genome-wide differential gene expression analysis. Our experimental design aimed to compare free-floating planktonic cells to adherent cells attached to either the unmodified or nanospiked Ti surfaces (Fig. 3). We identified 246 differentially expressed genes (DEGs) common to both comparisons (Supplementary Table 2), indicative of the phenotype switch from planktonic to adherent cells. More importantly, we identified a set of 87 DEGs unique to the comparison between unmodified Ti and planktonic cells, and a further 78 unique DEGs in the nanospiked Ti comparison (Supplementary Tables 3 and 4, respectively). From our differential gene expression analysis of *S. aureus* on the nanospiked and unmodified surfaces, we identified a set of uniquely upregulated genes associated with the biosynthesis of peptidoglycan and the modification of cell surface structures on the unmodified Ti surface (Table 1).

**Table 1:** Uniquely upregulated genes, from the unmodified Ti comparison, involved in the cell wall assembly, growth and division.

Genes associated with cell surface assembly and modification			
*Gene symbol*^a^, product	GeneID	Functional description	*P*
*Dat* D-amino-acid transaminase	KQ76_RS08950	Catalyses synthesis of d-glutamate and d-alanine for peptidoglycan synthesis	0.023
*murD* UDP-N-acetylmuramoyl-l-alanine--d-glutamate ligase	KQ76_RS05595	Catalyses the formation of the peptide bond between UDP-N-acetylehyl-l-alanine and d-glutamic acid	0.024
*alr* Alanine racemase	KQ76_RS06790	Converts between l-alanine to d-alanine	0.028
Polysaccharide biosynthesis protein	KQ76_RS08970	Biosynthesis of polysaccharides for peptidoglycan and capsule formation	0.046
Undecaprenyl/decaprenyl-phosphate alpha-N-acetylglucosaminyl 1-phosphate transferase	KQ76_RS03675	Involved in biosynthesis and organization of cell wall	0.036
*isaA* lytic transglycosylase IsaA	KQ76_RS13190	Hydrolysis of peptidoglycan during cell growth and division	0.042
*lytM* glycine-glycine endopeptidase LytM	KQ76_RS01125	Hydrolysis of peptidoglycan during cell growth and division	0.029
amidase domain-containing protein	KQ76_RS13615	Hydrolysis of peptidoglycan during cell growth and division	0.024
CHAP domain-containing protein	KQ76_RS01150	Hydrolysis of peptidoglycan during cell growth and division	0.038
amidohydrolase family protein	KQ76_RS13245	Hydrolysis of peptidoglycan during cell growth and division	0.049
*hom* homoserine dehydrogenase	KQ76_RS06420	Coordination of a metabolic pathway that leads to the synthesis of cell-wall components such as l-lysine and m-DAP, as well as other amino acids such as l-threonine, l-methionine, l-isoleucine	0.030
*dltB* PG: teichoic acid d-alanyltransferase DltB	KQ76_RS04160	d-alanylation of teichoic acids	0.020
*dltD* d-alanyl-lipoteichoic acid biosynthesis protein DltD	KQ76_RS04170	d-alanylation of teichoic acids	0.032
*tarS* poly(ribitol-phosphate) beta-N-acetylglucosaminyltransferase	KQ76_RS01020	β-O-GlcNAcylation of teichoic acids	0.034
*mprF* bifunctional lysylphosphatidylglycerol flippase/synthetase MprF	KQ76_RS06595	Lysylation of phosphatidylglycerol	0.023

All genes in the table were uniquely upregulated on the unmodified Ti surface, but not the nanospiked surface. The table includes gene symbols where available (italics), product name (as described by NCBI), GeneID (unique to NCBI reference genome database), functional description, and *P*-values.

^a^Gene symbols are provided for those which are specifically labelled in the reference genome of ATCC25923 on NCBI.

**Fig. 4:**
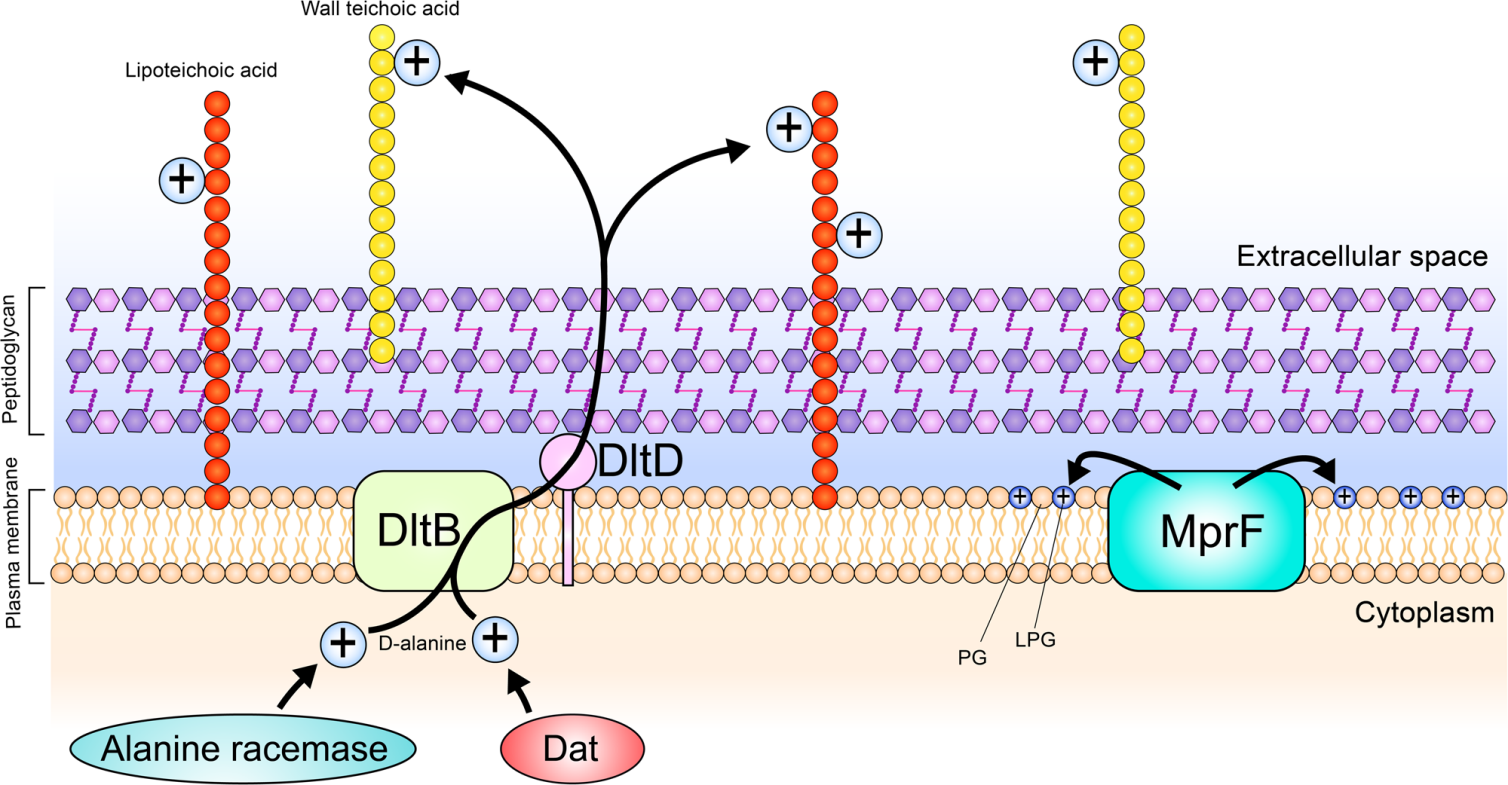
Genes involved with the modification of cell wall surface charge of *S. aureus*, which was uniquely upregulated on the unmodified Ti surface. The positively charged d-alanine is generated in the cytoplasm by the actions of d-amino acid transferase (Dat) and alanine racemase. The newly synthesized d-alanine is then brought across the plasma membrane and covalently attached to teichoic acids by the two membrane-bound proteins DltB and DltD. This modification reduces the overall negative charge associated with teichoic acids. Another membrane-bound protein, multiple peptide resistance factor (MprF), converts phosphatidylglycerol (PG) into lysyl-phosphatidylglycerol (LPG) and then orients the modified phospholipid to sit within the outer leaflet of the phospholipid bilayer using its flippase activity. Through this bifunctional activity, MprF also contributes to the reduction of the overall negative charge of the *S. aureus* cell surface.

From the DEGs listed in Table 1, we identified a group of genes associated with the modification of cell surface charge (Fig. 4). Expression of these genes was upregulated on the unmodified Ti surface in comparison to the nanospiked surface.

### Nanospike-induced changes in *S. aureus* cell biochemistry

**Fig. 5:**
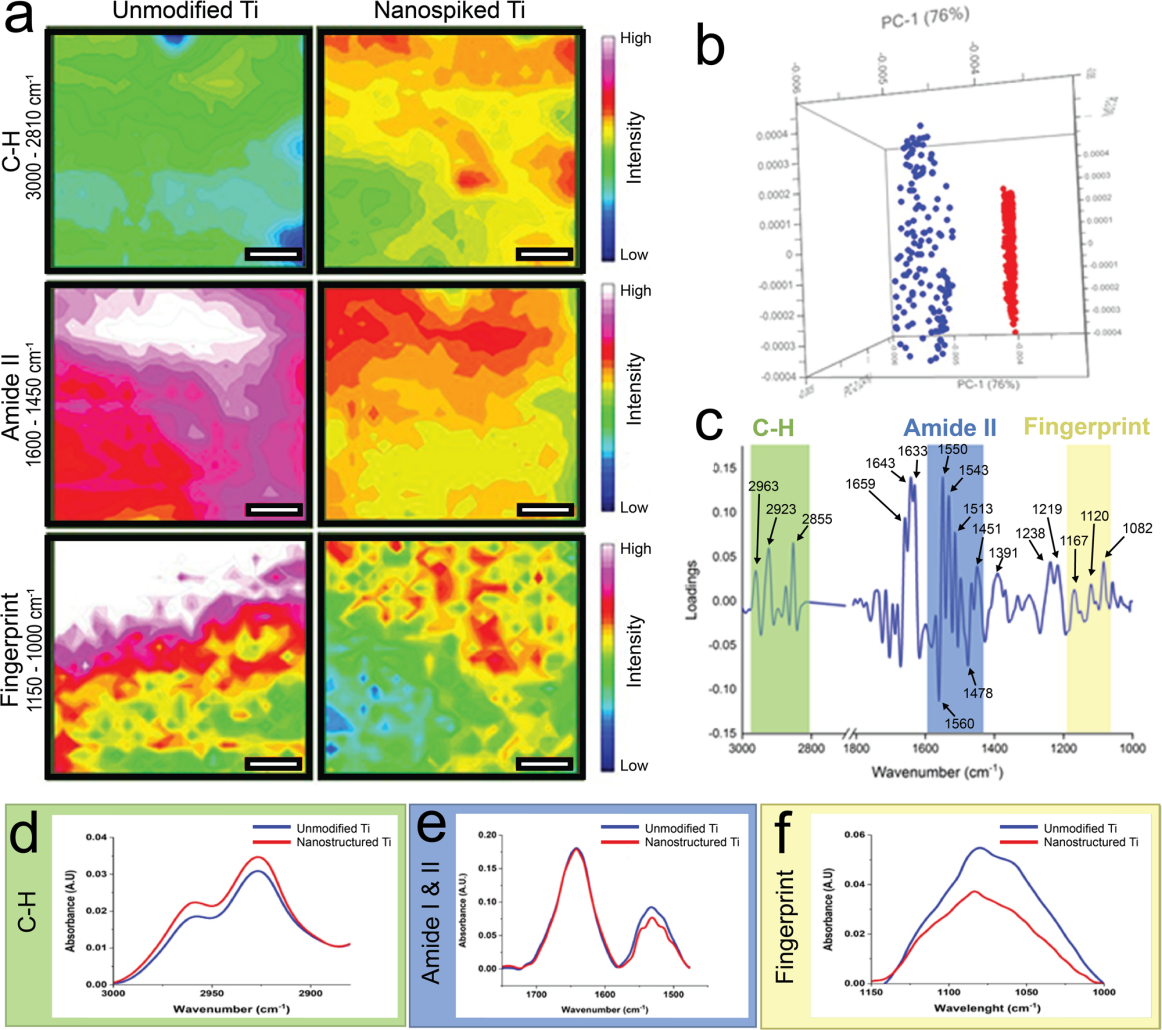
Synchrotron ATR-FTIR microspectroscopy of *S. aureus* attached to a nanospiked titanium surface. **a** Heat maps showing the distribution of normalized absorption intensity of the key biochemical components (i.e. lipids, proteins, and polysaccharides/nucleic acids). Scale bars are 5 µm. **b** PCA scores plots of *S. aureus* cells incubated on unmodified and nanospiked samples, showing the separation of cells on unmodified Ti (blue) and cells on nanospiked Ti (red) into two clusters. **c** Corresponding PCA loadings plot, indicating the three key biochemical components in *S. aureus* that influenced the separation of the cells on unmodified and nanospiked Ti samples. **d–f** Comparisons of the average spectra extracted from hierarchical cluster analysis for *S. aureus* in three key spectral regions that represent lipids, proteins, and nucleic acids, respectively.

To further investigate the biochemical changes in *S. aureus* induced by the titanium nanospikes, we utilized synchrotron ATR-FTIR microspectroscopy^19^ (Fig. 5). Three spectral regions containing the key biochemical information were analysed. A flowchart of the analysis is presented in Supplementary Fig. 3. The ν(C-H) stretching modes observed in the 3000–2850 cm^−1^ spectral range represent mainly lipid composition in the cells, while the amide I and II bands in the 1650–1450 cm^−1^ range are attributable to secondary protein conformation. The spectral features in the 1150–1000 cm^−1^ range involve vibrational modes related to nucleic acids and polysaccharides^20^. Heatmaps for the Amide 1 region were captured to confirm the presence of bacteria in the area scans (Supplementary Fig. 4). The heatmaps presented in Fig. 5a show the normalized absorption intensity of these key biochemical components in three spectral regions, revealing their spatial distribution across the cell-substrate interface. Differences in all 3 regions were observed. While the differences observed in the spectral maps are qualitatively overt, their statistical significance was confirmed using hierarchical cluster analysis (HCA) to group spectra with similar spectral features. Following this, variations in chemical interactions of cells on unmodified and nanospiked Ti were assessed by principal component analysis (PCA) using principal component 1 (PC-1, Fig. 5b). The differences between the unmodified and nanospiked Ti groups were largely described by PC-1 (76%). The clustering and distribution of scores between unmodified and nanospiked samples in the PCA plot indicate an unambiguous difference between the two sample types. The PC-1 loadings plot (Fig. 5c) was chosen in this study to represent the major biochemical changes of *S. aureus*, which were influenced by the nanospiked Ti. This enabled the identification of absorption peaks, representing significant chemical differences. To appreciate the overall magnitude of difference in the chemical signatures identified in the PCA loading peaks, average spectra were generated for the 3 regions (Fig. 5d–f) (3000–2850, 1650–1450, and 1150–1000 cm^−1^). The most profound change was noted in the regions associated with lipids, polysaccharides and nucleic acids. A lesser degree of change was also noted for the spectral range associated with protein, and this was almost exclusively in the Amide II band of the spectrum (1550–1500 cm^−1^), potentially indicating changes in protein conformation^21^.

## Discussion

We set out to perform an in-depth molecular and biochemical characterization of *S. aureus* on a nanospiked surface and elucidate potential mechanisms underpinning surface-induced antibiotic sensitization. The nanospiked surface was prepared by hydrothermal etching as previously described^22^, due to well-established bactericidal efficacy^23^. Material and chemical characterization of the nanospiked Ti surface is presented in the supplementary information (Supplementary Fig. 1, Supplementary Table 1). In the present study, we noted that the bactericidal activity of the nanospikes is not an instantaneous process but instead occurs cumulatively over time. This is most evident from the Live/Dead staining assay, which shows a progressive increase in red-stained cells over 24 h. This is also further evidenced by colony enumeration (Supplementary Fig. 7). When cultured on the unmodified Ti, the number of viable cells retrieved from the surface exponentially increased by ~4 logarithms over 24 h. This is in contrast to the nanospiked Ti group, which showed a more gradual accumulation of cells, and almost 2 logarithms fewer viable cells compared to the unmodified Ti surface at 24 h. The rate of *S. aureus* biovolume growth on the nanospiked surface was impeded, but the rate of EPS secretion was approximately equal between both sample types. However, while the EPS on the unmodified Ti surface tended to accumulate around cell clusters, it was more homogenously distributed across the entire surface of the nanospiked Ti. This is plausibly a stress response effect, considering that *S. aureus* has been reported to manipulate its EPS secretions as a response to mechanical stress^24^. We aimed to investigate the change in vancomycin tolerance of early-adherent *S. aureus* cells on both surface types. Interestingly, a mere 3 h of attachment to the unmodified surface was sufficient to enable *S. aureus* to persist through vancomycin treatment, and this effect was strengthened with 6 h of pre-treatment attachment. Conversely, on the nanospiked Ti surface, *S. aureus* was not able to persist through vancomycin treatment irrespective of pre-treatment attachment duration. Interestingly, the summative bactericidal effect of the nanospikes and vancomycin led to total clearance of *S. aureus* even when the vancomycin concentration was reduced to 0.5 × MIC (Supplementary Fig. 5). This indicates that the synergistic effects of vancomycin and the nanospikes provide a comparatively greater bactericidal effect than the antibiotic alone. This raises the question, how does the nanospiked Ti surface influence the antibiotic tolerance of *S. aureus*? Previous reports have identified various mechanisms of antibiotic defence in adherent bacterial cells, but this concept is usually discussed in the context of established biofilms. A commonly cited drug tolerance factor related to biofilms is the secretion of EPS and an associated restriction of antibiotic penetration^25^. From our measurements, EPS secretions were equal between surface types throughout the study. Due to this, the degree of EPS secretion can be reliably ruled out as the differentiating factor. Although there was an overall increase in biovolume on the unmodified Ti surface over 24 h, in the first 6 h of attachment, there was no significant difference between surface types. As we see a substantial difference in antibiotic tolerance during the first 6 h, the evidence suggests that biovolume is not the differentiating factor between sample types. To further investigate differences in factors associated with antibiotic defence, we performed a differential gene expression between planktonic cells and cells attached to either the unmodified or nanospiked surface. Broadly, the cells incubated on the unmodified surface showed an expression profile that reflects a comparatively greater aptitude to assemble and remodel peptidoglycan and its associated surface structures. The upregulation of *dltB* and *dltD* on *S. aureus* cells incubated on the unmodified Ti promotes the d-alanylation of teichoic acids to reduce the net negative charge of the cell surface^26^. In parallel, the upregulation of *dat*^27^ and alanine racemase^28^ fuels the production of D-alanine required for this modification. Similarly, the increased expression of *mprF* enables the cells on the unmodified surface to produce the positively charged phospholipid LPG^29^, to further reduce the net negative charge on the outer surface of the cell. To obtain further insight, we used ATR-FTIR to compare the biochemical signatures of cells attached to both types of surfaces. Supporting evidence for differences in d-alanylation of teichoic acids and lysylation of PG was found by identifying spectral peaks (Supplementary Table 5) associated with primary amines (present in LPG) or esters (present in d-alanylated teichoic acids). The FTIR spectra for both chemical markers were higher in cells on the unmodified Ti surface. Interestingly, on the unmodified surface, we observed a large increase of the bands in the region of 1180–1000 cm^−1^. This region captures both polysaccharides and nucleic acids. Due to the similarity between the spectra of nucleic acids and polysaccharides, the FTIR spectra must be corroborated with other data to establish a biological basis for the measurements. In our gene expression analysis, we reported an upregulation of a polysaccharide biosynthesis protein (KQ76_RS08970, Supplementary Table 3) which was uniquely upregulated on the unmodified surface. This suggests that the increased intensity of the IR spectra in this range is at least partially attributable to increased biosynthesis of polysaccharides. These data suggest that cells on the unmodified surface have a greater capacity to form peptidoglycan and modify the charge-bearing structures on both the cell wall and membrane bilayer. The modification of cell surface charge via d-alanylation of teichoic acids^30^ and lysylation of PG^31^ has been recognized as an important virulence factor in *S. aureus*^32^. We propose that the modification of cell surface charge is the primary differentiating factor that enables *S. aureus* to persist through vancomycin treatment on an unmodified Ti surface. This is because a reduction in net negative surface charge enables the cell to repel positively charged compounds, such as cationic antimicrobial peptides and some antibiotics including daptomycin and vancomycin^33^. In this way, the nanospikes nullify one of the main mechanisms of vancomycin tolerance in *S. aureus*. It is presently unclear exactly what triggers the differential expression of genes associated with cell surface structures, but a clue for this may be found in the accumulation of inorganic phosphate (P_i_). It has previously been shown that when *S. aureus* accumulates P_i_, the *dlt* operon becomes upregulated, leading to increased d-alanylation of teichoic acids and a greater tolerance to the antibiotic daptomycin^33^. Our results showed an increased expression of a P_i_ transporter (KQ76_RS03230, Supplementary Table 3) on the unmodified Ti surface, enabling *S. aureus* to better accumulate P_i_ from its surroundings.

Our findings have major implications for the biomedical field for multiple reasons. Firstly, we have provided a detailed mechanistic insight into how nanospiked surfaces can sensitize bacteria to the positively charged antibiotic vancomycin. This occurs in the very early stages of cell attachment. These findings are relevant to many implant surgical procedures, where a prophylactic dose of vancomycin is administered to the patient prior to implant placement. This is impactful because the majority of implant infections arise from contamination during surgical placement^34^. As the hostile effects of the nanospiked surface are mediated by mechanical forces^35^ rather than chemical ones, this synergy can be considered substrate-ambivalent. As such, similar nanoengineered structures can be translated beyond titanium and its alloys, to many other material types such as silicon or PMMA^36^, meaning this synergistic interaction can be harnessed in many more implant applications. These findings support and further explain our previous finding, related to a late-onset infection scenario, that established biofilms could be completely eradicated with sub-clinical concentrations of antibiotics when incubated on a nanospiked surface^37^. It is now evident that the synergy between mechanical stress and antibiotic treatment could benefit patients at the time of implant placement thus preventing biofilm formation and infection from occurring. Furthermore, as we observed a similar synergistic effect at 0.5 × MIC, this suggests that clinicians may be able to reduce antibiotic dosages administered to patients to curb the rate of nephrotoxicity^38^. While these in vitro results are highly encouraging, it must be noted that the nature of the in vitro experimental design has limitations. To address this, an appropriate in vivo model must be designed and implemented to investigate whether the presence of host proteins and immune cells may affect the synergy between bactericidal nanostructures and vancomycin treatment. Furthermore, the interaction described in the present report falls into the category of antibiotic tolerance. The described antimicrobial synergy is unlikely to apply in cases where the pathogen possesses specific antibiotic resistance genes such as the *van* cluster of genes responsible for vancomycin resistance^39^.

## Methods

### Cultures and conditions

*Staphylococcus aureus* ATCC 25923 was retrieved from glycerol stocks kept at −80 °C, and incubated on Tryptone Soy Agar (TSA, Oxoid, Thermo Fisher, MA) overnight at 37 °C. A single colony was aseptically transferred to a 5 mL tube containing Tryptone Soy Broth (TSB, Oxoid, Thermo Fisher, MA) and incubated until the late log phase. Cell density was determined by optical density at 600 nm (OD_600_) using the cuvette reader of a Nanodrop 2000 (Thermo Scientific, MA, USA).

### Surface inoculation

*S. aureus* pre-culture was diluted to OD_600_ = 0.1, which is equivalent to approximately 10^8^ cfu/mL. Titanium samples were placed into sterile 24-well plates and immersed in 1 mL of the diluted *S. aureus* culture. The plates were incubated in an opaque humid chamber (a sealed plastic box with a damp paper towel) at 37 °C on an orbital shaker (Ratek Instruments Pty. Ltd., VIC, Australia) at 90 RPM.

### Live/Dead and biovolume analysis

Following incubation, samples were transferred to sterile 24-well plates and immersed in BacLight Live/Dead reagent (Invitrogen, MA, USA), prepared with equal concentrations of SYTO9 and Propidium Iodide at 1.5 µL/mL phosphate-buffered saline (PBS). The samples were incubated for 15 min in the dark and then imaged with an Olympus FV3000 confocal laser scanning microscope (CLSM, Olympus, Tokyo, Japan). Excitation and emission spectra were set to 480/500 for SYTO9 and 490/635 for Propidium Iodide, as instructed by the manufacturer. Micrographs were taken at 40x magnification at 3 random locations per sample. For viability analysis, proportions of green and red cells were counted in ImageJ v1.53a (NIH, Bethesda, MD, USA). To enumerate the cells, micrographs were split into individual colour channels (red, green and blue) in ImageJ. The ‘find maxima’ tool was used to quantify the number of spots appearing in the green and red channels. Viability was then calculated with the following equation: ${\rm{Viability}} = \frac{{{\rm{green}}\,{\rm{cells}}}}{{{\rm{total}}\,{\rm{cells}}}} \times 100$. For biovolume measurements, 3D Z-stack images were taken in triplicate and analysed using the ‘surface’ function in Imaris 3D analysis software v9.3.0 (Bitplane, Zürich, CHE).

### EPS staining with FilmTracer™ SYPRO™ Ruby

Samples were immersed in SYPRO Ruby (Invitrogen, MA, USA) as directed by the manufacturer. Following incubation, samples were imaged with an Olympus FV3000 CLSM, using the excitation and emission spectra of 450/610. Triplicate micrographs were taken at ×40 magnification. The fluorescence intensity of EPS was quantified in ImageJ v1.53 and normalized by the following procedure: normalized fluorescence of EPS (NFEPS) = integrated density−background fluorescence, where Integrated Density represents the total raw fluorescence per micrograph, and Background Fluorescence is the measurement of fluorescence obtained from unmodified or nanospiked Ti surfaces stained with SYPRO Ruby, without the presence of bacteria.

### Vancomycin tolerance of surface attached S. aureus

To determine the tolerance of adherent *S. aureus* to vancomycin (Merck, NJ, USA), we cultured *S. aureus* on the samples according to the inoculation procedure described above, for either 3 or 6 h. Following culture, we rinsed non-adherent cells from the Ti samples by gently dipping them in sterile PBS 3 times. We then immersed the Ti samples in TSB supplemented with vancomycin at a concentration equal to its MIC (1 µg/mL, Supplementary Fig. 6) for 24 h in a humid box at 37 °C on an orbital shaker set to rotate at 90 RPM. Post-treatment viability was determined following the BacLight LIVE/DEAD Bacterial Viability Kit (ThermoFisher, MA, USA) protocol described above.

### RNA extraction

*S. aureus* was cultured on titanium samples as previously described for 24 h, then cells were retrieved by vortex for 30 s followed by ultrasonication for 2 mins. Cell concentrations were measured and then normalized by dilution with sterile PBS. Cells were pelleted and resuspended in the lysis buffer provided in the RiboPure Bacterial RNA extraction kit (Invitrogen, MA, USA), and RNA was extracted and isolated following the directions of the manufacturer. We utilized the RNA TapeStation 2200 (Agilent, CA, USA) to evaluate RNA quality and the Qubit (Thermo Fisher, MA, USA) for quantification. Libraries were generated following the manufacturer’s instructions using the NuGEN Universal Prokaryotic RNA-seq kit (NuGEN, CA, USA) and included 13 cycles of amplification.

### RNA sequencing

An MGI DNBSEQ G400 was equipped with a PE100 flow cell (MGI Tech Co., Ltd, Shenzhen, China), and used for high throughput sequencing of *S. aureus* RNA samples. PolyA libraries were prepared using Tecan Universal Prokaryotic RNA-seq (Tecan Group Ltd, Männedorf, Switzerland). Conversion from Illumina to MGI Library was performed with MGIEasy Universal Library Conversion Kit (Part No. MGI1000004155, MGI Tech Co., Ltd, Shenzhen, China). RNA-seq data pre-processing was done with an in-house pre-processing workflow, using MultiQC for quality reporting, STAR for alignment to the *S. aureus* genome assembly (GCA_000756205.1), and FeatureCounts for quantification of gene expression. There was an 85% alignment and library sizes were over 12 M/sample, ensuring their suitability for downstream analysis in R. Limma-Voom (v.3.52.0) was used for the analysis of differential gene expression. Two different comparisons were used to observe the differential expression of genes (DEG) across three conditions (free-floating planktonic cells vs. cells adherent to either unmodified or nanospiked Ti. Significant DEGs were identified for each comparison (FDR-adjusted *P*-value < 0.05).

### Synchrotron ATR-FTIR microspectroscopy

*S. aureus* ATCC25923 cells were grown in TSB until the late log phase and diluted to OD_600_ = 0.1 (10^8^ CFU/mL). The cells were analysed at the interface of the nanospiked Ti surface using synchrotron ATR-FTIR microspectroscopy at the Infrared Microspectroscopy (IRM) beamline, ANSTO—Australian Synchrotron (Victoria, Australia). The synchrotron ATR-FTIR experiment was performed using a Bruker Vertex 80v spectrometer coupled with a Hyperion 3000 FTIR microscope and a liquid nitrogen-cooled narrow-band mercury cadmium telluride (MCT) detector (Bruker Optik GmbH, Ettlingen, Germany). All the synchrotron ATR-FTIR spectra were recorded within a spectral range of 3900‒750 cm^−1^ using 4-cm^−1^ spectral resolution. Blackman-Harris 3-Term apodization, Mertz phase correction, and zero-filling factor of 2 were set as default acquisition parameters using OPUS 8.0 software suite (Bruker Optik GmbH, Ettlingen, Germany).

*S. aureus* ATCC25923 cells exposed to two types of Ti surfaces were subsequently analysed and imaged in macro ATR-FTIR mapping mode, using an in-house developed macro ATR-FTIR device equipped with 250-μm-diameter facet germanium (Ge) ATR crystal (*n*_Ge_ = 4.0), and a ×20 IR objective (NA = 0.60; Bruker Optik GmbH, Ettlingen, Germany)^19^. The unique combination of the high refractive index of the Ge ATR crystal and the high numerical aperture (NA) objective used in this device, when coupled to the synchrotron-IR beam, allowed the surface characterization of the concentrated microbial cell samples to be performed at a high spatial resolution using <1 µm step interval. The fixed cell samples were then placed into the sample stage of the macro ATR-FTIR unit. After that, the Ge ATR crystal was brought to the focus of the synchrotron-IR beam, and a background spectrum was recorded in the air using 4 cm^−1^ spectral resolution and 256 co-added scans. Consecutively, the samples were brought into contact with the sensing facet of the Ge ATR crystal, and a synchrotron macro ATR-FTIR spectral map was acquired. Chemical maps were generated from the embedded spectra by integrating the area under the relevant peaks using the OPUS 8.0 software.

Multivariate data analysis including hierarchical cluster analysis (HCA) and principal component analysis (PCA) was performed using CytoSpec v. 1.4.02 (Cytospec Inc., Boston, MA, USA) and The Unscrambler X 11.1 software package (CAMO Software AS, Oslo, Norway), respectively. HCA was carried out in the form of vector-normalized 2nd derivative spectra using Ward’s algorithm and five clusters within two spectral regions (i.e. 3004–2800 and 1800–1000 cm^−1^). These specific spectral ranges contain the molecular information most relevant to the microbial samples, particularly lipids, proteins, polysaccharides and nucleic acids. The selected clusters from the HCA were subsequently imported into the Unscrambler X 11.1 software to proceed with PCA. After that, PCA was performed on the combined datasets of unmodified and nanospiked Ti samples in the form of EMSC-corrected 2nd derivative spectra, in order to investigate similarities and differences between the two cell groups. It should be emphasized that the Savitzky–Golay algorithm removes the broad baseline offset and curvature, whilst the extended multiplicative scatter correction (EMSC) algorithm removes light scattering artefacts and normalizes the spectra accounting for path length differences. As a result, the EMSC-corrected 2nd derivative spectra respond more linearly to the analyte concentration when compared to those obtained from untreated spectra, leading to better interpretability, more robust calibration models, and thereby an improved predictive accuracy^20^. The PCA results containing scores and loadings plots from the first three principal components (PCs) were used for analysis to identify the key biochemical components influencing the differentiation between cells exposed to the unmodified and nanospiked Ti samples.

### Statistics

Graphical data is represented with mean and standard deviation and plotted with GraphPad Prism v9.0.0 (GraphPad Software, CA, USA). A two-way ANOVA with post hoc analysis using the Bonferroni method of multiple comparisons was used to measure statistical significance. To analyse the gene expression data, limma-voom moderated *t*-test was used^40^. Significance was defined as a *P*-value < 0.05. All experiments were performed in triplicate, except for the RNA sequencing experiment in which we used four replicates.

### Reporting summary

Further information on research design is available in the Nature Research Reporting Summary linked to this article.

### Supplementary information

Supporting Info

Reporting Summary

## Data Availability

The raw RNA-seq data used in this study were deposited in NCBI Sequence Read Archive, with accession number PRJNA986675.
