# Supplementary material for: Vancomycin tolerance of adherent Staphylococcus aureus is impeded by nanospike-induced physiological changes
Source: NPJ Biofilms Microbiomes. 2023 Nov 29;9:90. doi: 10.1038/s41522-023-00458-5 (PMC10687013; doi:10.1038/s41522-023-00458-5)
Supplement: Supplementary file 1 — Supporting Info [file 41522_2023_458_MOESM1_ESM.pdf]

Vancomycin Tolerance of Adherent *Staphylococcus aureus* is Impeded by Nanospike-induced Physiological Changes

Andrew Hayles[1], Richard Bright[1], Ngoc Huu Nguyen[2], Vi Khanh Truong[1], Jonathan Wood[3], Dennis Palms[1], Jitraporn Vongsvivut[4], Dan Barker[5], Krasimir Vasilev[1*]

[1] College of Medicine and Public Health, Flinders, University, Bedford Park 5042, South Australia, Australia

[2] School of Biomedical Engineering, Faculty of Engineering, University of Sydney, Sydney, Australia

[3] Academic Unit of STEM, University of South Australia, Mawson Lakes, Adelaide, 5095, South Australia, Australia.

[4] Infrared Microspectroscopy (IRM) Beamline, ANSTO – Australian Synchrotron, 800 Blackburn Road, Clayton, Victoria 3168, Australia

[5] Corin Australia, Baulkham Hills, New South Wales 2153, Australia

(* Corresponding author: Krasimir.vasilev@flinders.edu.au)

Keywords: gene expression, nanospikes, biofilm, syrface charge, biomaterials, synchrotron, ATR-FTIR,

Table of Contents:

**Supplementary Figure 1**	Physical and chemical characterization of nanostructured titanium	**3**
**Supplementary Table 1**	Surface roughness and area measurements of unmodified and nanospiked Ti surfaces	**4**
**Supplementary Figure 2**	The chemical structure, molecular mass and formal molecular charge of vancomycin at physiological pH.	**4**
**Supplementary Table 2**	Common DEGs identified in both the planktonic vs unmodified and nanospiked Ti comparisons	**5**
**Supplementary Table 3**	Unique DEGs identified in the planktonic vs unmodified Ti comparison.	**11**
**Supplementary Table 4**	Unique DEGs identified in the planktonic vs nanospiked Ti comparison	**13**
**Supplementary Table 5**	Summary of relevant *S. aureus* cell components and products, along with their chemical bond markers and associated spectral regions	**16**
**Supplementary Figure 3**	The flow of analysis used to generate and process ATR-FTIR data	**17**
**Supplementary Figure 4**	Heatmaps of the Amide I region on umodified and nanospiked Ti	**17**
**Supplementary Figure 5**	Activity of sub-MIC dose vancomycin against *S. aureus* attached to the nanospiked surfaces for the increasing duration	**18**
**Supplementary Figure 6**	Minimum inhibitory concentration of vancomycin against *S. aureus* ATCC25923	**18**
**Supplementary Figure 7**	Colony enumeration over 24 h on Unmodified Ti and Nanospiked Ti	**19**

Results

Characterization of nanostructured titanium

We fabricated the nanospiked Ti surface using an alkaline hydrothermal etching method previously described.[1] Material characterization was carried out to verify the consistency of the method (Fig. S1 and Table S1). We found a mean spike height of 306 ± 78 nm, and width of 75 ± 21 nm, which is consistent with previously published results. The mean roughness, as measured by arithmetic average (Ra) was 10.1 nm on the unmodified Ti, and 61.5 nm on the nanospiked Ti surface. EDS analysis showed a decrease in the proportion of Ti and an increase in O on the nanospiked surface, reflecting the oxidation of Ti resulting from the hydrothermal procedure. The change in surface wettability was measured using water contact angle, which showed a decrease from 40° to <10° on the nanospiked Ti surface. This indicates a substantial increase in the hydrophilicity of the surface.

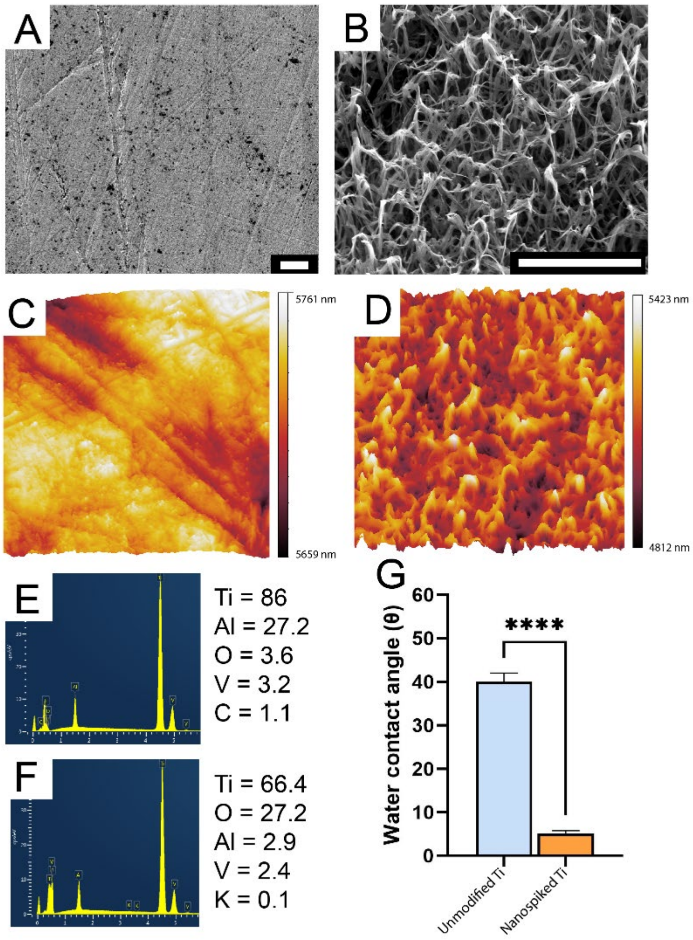

Supplementary Figure 1. Physical and chemical characterization of nanostructured titanium. A and B) SEM micrographs of the unmodified and nanospiked Ti surfaces, respectively. C and D) AFM images of unmodified and nanospiked Ti surfaces, showing differences in their surface roughness. E and F) EDS measurements of the surface of unmodified and nanospiked Ti. G) Comparison of water contact angles observed for unmodified and nanospiked Ti surfaces. **** $P < 0.0001$, n=3, mean ± *SD*.

Supplementary Table 1. Surface roughness and area measurements of unmodified and nanospiked Ti surfaces.

Measurement	Unmodified Ti	Nanospiked Ti
Arithmetic average (Ra), nm	10.1	61.5
Root mean square (RMS), nm	6.6	88.5
Surface Area, μm^2	25.2	50.3

Molecular properties of vancomycin

We aimed to measure the influence of the molecular charge of vancomycin on its efficacy against adherent *S. aureus* attached to both surface types. Vancomycin is a glycopeptide antibiotic that binds to the D-Ala-D-Ala residues on newly forming peptidoglycan sheets, preventing crosslinking from occurring.[2] Vancomycin was chosen due to its clinical relevance in prophylactic treatments during implant placement (Fig S2). The charge of vancomycin at physiological pH was calculated using the Henderson-Hasselbalch equation, with pKa values for titratable functional groups of vancomycin obtained in the relevant literature.[3]

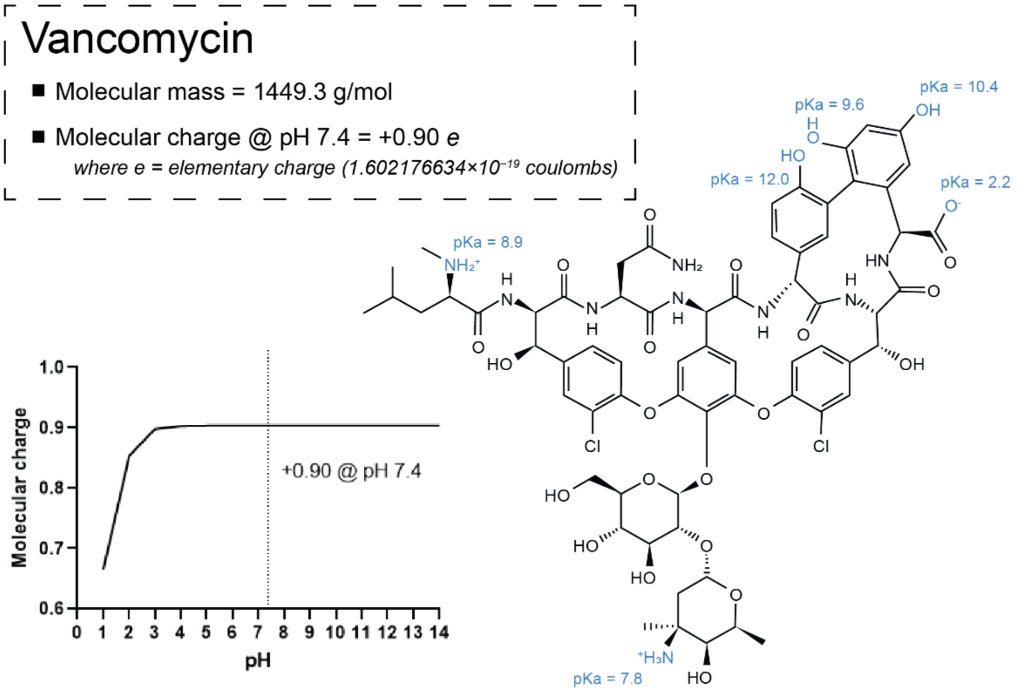

Supplementary Figure 2. The chemical structure, molecular mass and formal molecular charge of vancomycin at physiological pH.

Calculating the molecular charge of vancomycin

The charge of both antibiotics was calculated as a function of pH, using the Henderson-Hasselbalch equation, with pKa values for the titratable groups obtained from relevant literature.[3]

$$pH = pKa + \log_{10}\frac{[A-]}{[HA]}$$

Equation 1. The Henderson-Hasselbalch equation. K_a is the dissociation constant of the weak acid, pK_a = -log K_a and $[HA]$ and $[A\text{-}]$ are the molar concentrations of the weak acid and its conjugate base.[4]

For each of the empirically determined titratable groups on the antibiotics, the degree of dissociation was calculated by inputting the pK_a and pH values. To determine the overall molecular charge at each pH level, the degrees of dissociation for each titratable group, along with their contribution to charge, were combined to generate a net molecular charge. An arbitrary example is given below for two titratable groups at pH 10:

Titratable group	*pKa*	*Degree of dissociation*	*Contribution to charge*
R-COOH	1.9	0.9999	0 → –
R-$NH3^+$	8.35	0.9781	+ → 0

In this arbitrary example, the overall molecular charge at pH 10 would be calculated by the following:

$$\text{Charge} = (-1 \times 0.9999) + (1 \times [1 - 0.9781]) = -0.978$$

Equation 2. An example of the calculation used to obtain molecular charge values using the degree of dissociation of each titratable group in a molecule.

Differential gene expression of S. aureus adherent on unmodified or nanospiked Ti

Supplementary Table 2. Common DEGs identified in both the planktonic vs unmodified and nanospiked Ti comparisons. GeneIDs are specific to the *S. aureus* ATCC25923 reference genome, as listed on the NCBI database. Gene symbols are provided where available and were acquired from the annotated reference genome. DEGs are sorted by log fold-change, where a positive value corresponds to an upregulation on HTE-Ti compared to planktonic.

**GeneID**	**Symbol**	**Product Description**	**Log FC**
KQ76_RS04860	sspB	cysteine protease staphopain B	5.875099
KQ76_RS01800		hypothetical protein	4.838287
KQ76_RS00865		3-hydroxyacyl-CoA dehydrogenase/enoyl-CoA hydratase family protein	4.677278
KQ76_RS04855	sspC	staphostatin B	4.58259
KQ76_RS00870		acyl-CoA dehydrogenase family protein	4.510791
KQ76_RS13580	aur	zinc metalloproteinase aureolysin	4.488095
KQ76_RS04300		argininosuccinate synthase	4.20662

KQ76_RS10335		cyclic lactone autoinducer peptide	4.038172
KQ76_RS00875		acyl--CoA ligase	3.728256
KQ76_RS04295	argH	argininosuccinate lyase	3.667964
KQ76_RS05000	purN	phosphoribosylglycinamide formyltransferase	3.470311
KQ76_RS00860		thiolase family protein	3.424063
KQ76_RS09170		excalibur calcium-binding domain-containing protein	3.362537
KQ76_RS07605		hypothetical protein	3.342676
KQ76_RS11430	rplX	50S ribosomal protein L24	3.234334
KQ76_RS08440		DUF4930 family protein	3.012092
KQ76_RS04440		ATP-binding cassette domain-containing protein	2.975198
KQ76_RS07600		hypothetical protein	2.956842
KQ76_RS11440	rpsQ	30S ribosomal protein S17	2.918199
KQ76_RS11475	rplW	50S ribosomal protein L23	2.914579
KQ76_RS06235		MerR family transcriptional regulator	2.913718
KQ76_RS01795		L-cystine transporter	2.84513
KQ76_RS06785		amidohydrolase	2.83644
KQ76_RS11435	rplN	50S ribosomal protein L14	2.796281
KQ76_RS11425	rplE	50S ribosomal protein L5	2.760127
KQ76_RS04400		MAP domain-containing protein	2.734562
KQ76_RS01155	esxA	WXG100 family type VII secretion effector EsxA	2.622365
KQ76_RS10750	atpE	F0F1 ATP synthase subunit C	2.618355
KQ76_RS09985		staphostatin A	2.612195
KQ76_RS13925		S8 family serine peptidase	2.606231
KQ76_RS13920		SAR2788 family putative toxin	2.597913
KQ76_RS06770	dapA	4-hydroxy-tetrahydrodipicolinate synthase	2.593283
KQ76_RS11465	rpsS	30S ribosomal protein S19	2.563046
KQ76_RS10185		hypothetical protein	2.550667
KQ76_RS11450	rplP	50S ribosomal protein L16	2.531738
KQ76_RS04995	purM	phosphoribosylformylglycinamidine cyclo-ligase	2.527018
KQ76_RS08790		alanine--glyoxylate aminotransferase family protein	2.493834
KQ76_RS11445	rpmC	50S ribosomal protein L29	2.481996
KQ76_RS06780	dapD	2,3,4,5-tetrahydropyridine-2,6-dicarboxylate N-acetyltransferase	2.479461
KQ76_RS11460	rplV	50S ribosomal protein L22	2.440601
KQ76_RS05625		cell division protein SepF	2.430133
KQ76_RS06455		catalase	2.399397
KQ76_RS05955	hslV	ATP-dependent protease subunit HslV	2.397299
KQ76_RS11115		YjiH family protein	2.389067
KQ76_RS05680		aspartate carbamoyltransferase catalytic subunit	2.363472
KQ76_RS06925		PTS glucose transporter subunit IIA	2.359194
KQ76_RS05685		dihydroorotase	2.34079
KQ76_RS07290	lukS-PV	Panton-Valentine bi-component leukocidin subunit S	2.288209
KQ76_RS04445		peptide ABC transporter substrate-binding protein	2.286407
KQ76_RS04965	purK	5-(carboxyamino)imidazole ribonucleotide synthase	2.28384
KQ76_RS02780		hypothetical protein	2.273974
KQ76_RS07020		dynamin family protein	2.261298

KQ76_RS04435		ABC transporter ATP-binding protein	2.25412
KQ76_RS11480	rplD	50S ribosomal protein L4	2.25045
KQ76_RS05965	codY	GTP-sensing pleiotropic transcriptional regulator codY	2.224011
KQ76_RS08795	serA	phosphoglycerate dehydrogenase	2.217932
KQ76_RS10755	atpB	F0F1 ATP synthase subunit A	2.197726
KQ76_RS11455	rpsC	30S ribosomal protein S3	2.185476
KQ76_RS05690		carbamoyl phosphate synthase small subunit	2.174814
KQ76_RS06540	acnA	aconitate hydratase AcnA	2.174178
KQ76_RS11420		type Z 30S ribosomal protein S14	2.17299
KQ76_RS11415	rpsH	30S ribosomal protein S8	2.170202
KQ76_RS04280		ornithine--oxo-acid transaminase	2.149646
KQ76_RS06680		Cof-type HAD-IIB family hydrolase	2.126842
KQ76_RS06820		acylphosphatase	2.122783
KQ76_RS13750	lip1	YSIRK domain-containing triacylglycerol lipase Lip1	2.119379
KQ76_RS04030		methionine ABC transporter ATP-binding protein	2.105125
KQ76_RS07685	proC	pyrroline-5-carboxylate reductase	2.095534
KQ76_RS08640	icd	NADP-dependent isocitrate dehydrogenase	2.036681
KQ76_RS08965		rRNA pseudouridine synthase	2.025482
KQ76_RS07970		deoxyribonuclease IV	1.970027
KQ76_RS11485	rplC	50S ribosomal protein L3	1.96629
KQ76_RS10770	upp	uracil phosphoribosyltransferase	1.963157
KQ76_RS11690	ureC	urease subunit alpha	1.953105
KQ76_RS07805	nusB	transcription antitermination factor NusB	1.940483
KQ76_RS11395	rpmD	50S ribosomal protein L30	1.928253
KQ76_RS04550		NAD kinase	1.890976
KQ76_RS05640		DivIVA domain-containing protein	1.86845
KQ76_RS11375	infA	translation initiation factor IF-1	1.847728
KQ76_RS00620		DUF2294 domain-containing protein	1.829735
KQ76_RS06930	msrB	peptide-methionine (R)-S-oxide reductase MsrB	1.828725
KQ76_RS08645		citrate synthase	1.769368
KQ76_RS04430		ABC transporter permease	1.760555
KQ76_RS06425	thrC	threonine synthase	1.756772
KQ76_RS06765		aspartate-semialdehyde dehydrogenase	1.737707
KQ76_RS10090		thioredoxin family protein	1.734299
KQ76_RS13450		CitMHS family transporter	1.734107
KQ76_RS04035		ABC transporter permease	1.7303
KQ76_RS08210	pxpA	5-oxoprolinase subunit PxpA	1.72953
KQ76_RS08675		NAD-dependent malic enzyme 4	1.725404
KQ76_RS05695	carB	carbamoyl-phosphate synthase large subunit	1.724676
KQ76_RS10450	leuB	3-isopropylmalate dehydrogenase	1.72296
KQ76_RS10445		2-isopropylmalate synthase	1.71414
KQ76_RS05945	trmFO	methylenetetrahydrofolate--tRNA-(uracil(54)- C(5))-methyltransferase (FADH(2)-oxidizing) TrmFO	1.711381
KQ76_RS08855	acsA	acetate--CoA ligase	1.677067
KQ76_RS06775	dapB	4-hydroxy-tetrahydrodipicolinate reductase	1.676859
KQ76_RS06240	glnA	type I glutamate--ammonia ligase	1.652739

KQ76_RS08890		hypothetical protein	1.634379
KQ76_RS01010		ribitol-5-phosphate dehydrogenase	1.61297
KQ76_RS08010		glycine--tRNA ligase	1.594851
KQ76_RS13590		hypothetical protein	1.593444
KQ76_RS11390	rplO	50S ribosomal protein L15	1.567387
KQ76_RS11400	rpsE	30S ribosomal protein S5	1.56473
KQ76_RS09095		CPBP family intramembrane metalloprotease	1.525028
KQ76_RS05675		NCS2 family nucleobase:cation symporter	1.504702
KQ76_RS08585	dnaI	primosomal protein DnaI	1.498073
KQ76_RS08665		acetyl-CoA carboxylase carboxyltransferase subunit alpha	1.481138
KQ76_RS07230	ebpS	elastin-binding protein EbpS	1.477509
KQ76_RS11410	rplF	50S ribosomal protein L6	1.464961
KQ76_RS08205		divalent metal cation transporter	1.45291
KQ76_RS07125		YpiB family protein	1.43023
KQ76_RS01185		TIGR04197 family type VII secretion effector	1.429717
KQ76_RS06370	cls	cardiolipin synthase	1.422521
KQ76_RS01160	esaA	type VII secretion protein EsaA	1.422495
KQ76_RS05610	ftsZ	cell division protein FtsZ	1.405476
KQ76_RS01165	essA	type VII secretion protein EssA	1.404665
KQ76_RS03105		glycosyltransferase family 2 protein	1.39927
KQ76_RS10130		aminotransferase class I/II-fold pyridoxal phosphate-dependent enzyme	1.38947
KQ76_RS08420	rplU	50S ribosomal protein L21	1.38866
KQ76_RS05950	xerC	tyrosine recombinase XerC	1.383844
KQ76_RS06430	thrB	homoserine kinase	1.380716
KQ76_RS07790		polyprenyl synthetase family protein	1.360145
KQ76_RS01450	glpT	glycerol-3-phosphate transporter	1.358195
KQ76_RS11380		adenylate kinase	1.35682
KQ76_RS07680		SDR family oxidoreductase	1.347439
KQ76_RS10425	ilvD	dihydroxy-acid dehydratase	1.329832
KQ76_RS02765		AMP-binding protein	1.314837
KQ76_RS06415		aspartate kinase	1.304811
KQ76_RS08215		acetyl-CoA carboxylase biotin carboxylase subunit	1.296582
KQ76_RS05670	pyrR	bifunctional pyr operon transcriptional regulator/uracil phosphoribosyltransferase PyrR	1.293504
KQ76_RS09930	gatB	Asp-tRNA(Asn)/Glu-tRNA(Gln) amidotransferase	1.281921
KQ76_RS07235		ATP-dependent DNA helicase	1.275003
KQ76_RS03350	mgrA	HTH-type transcriptional regulator MgrA	1.272681
KQ76_RS08865		acetoin utilization protein AcuC	1.253553
KQ76_RS04660		competence protein ComK	1.234448
KQ76_RS06435		Cof-type HAD-IIB family hydrolase	1.232642
KQ76_RS11775		2-hydroxyacid dehydrogenase family protein	1.232146
KQ76_RS07140	aroB	3-dehydroquinate synthase	1.223334
KQ76_RS10945	czrA	Zn(II)-responsive metalloregulatory transcriptional repressor CzrA	1.218973
KQ76_RS11405	rplR	50S ribosomal protein L18	1.216407

KQ76_RS05105	def	peptide deformylase	1.202483
KQ76_RS03825	gap	type I glyceraldehyde-3-phosphate dehydrogenase	1.201252
KQ76_RS05825	plsX	phosphate acyltransferase PlsX	1.193764
KQ76_RS02770		thiolase family protein	1.178227
KQ76_RS04955	folD	bifunctional methylenetetrahydrofolate dehydrogenase/methenyltetrahydrofolate cyclohydrolase FolD	1.173907
KQ76_RS06225	hflX	GTPase HflX	1.162707
KQ76_RS11685		urease subunit beta	1.155727
KQ76_RS05920	sucD	succinate--CoA ligase subunit alpha	1.126213
KQ76_RS07620		DUF1672 domain-containing protein	1.115425
KQ76_RS02975		hypothetical protein	1.108119
KQ76_RS05665		RluA family pseudouridine synthase	1.093772
KQ76_RS07975		DEAD/DEAH box helicase	1.087345
KQ76_RS11365	rpsM	30S ribosomal protein S13	1.082828
KQ76_RS10430	ilvB	biosynthetic-type acetolactate synthase large subunit	1.075309
KQ76_RS07670		aldo/keto reductase	1.06602
KQ76_RS07145	aroC	chorismate synthase	1.063711
KQ76_RS01790		NADPH-dependent oxidoreductase	1.049088
KQ76_RS08960		YtxH domain-containing protein	0.998704
KQ76_RS07570		hypothetical protein	0.997274
KQ76_RS04985	purL	phosphoribosylformylglycinamidine synthase subunit PurL	0.996956
KQ76_RS03670		GGDEF domain-containing protein	0.988453
KQ76_RS09805		aminopeptidase	0.983619
KQ76_RS04040		MetQ/NlpA family ABC transporter substrate-binding protein	0.960481
KQ76_RS00285		L-lactate permease	0.945314
KQ76_RS08050	floA	flotillin-like protein FloA	0.874902
KQ76_RS02595	rplL	50S ribosomal protein L7/L12	0.869492
KQ76_RS12715		AbgT family transporter	0.86198
KQ76_RS05830	fabD	ACP S-malonyltransferase	0.861921
KQ76_RS06555	plsY	glycerol-3-phosphate 1-O-acyltransferase PlsY	0.855538
KQ76_RS12230		NarK/NasA family nitrate transporter	0.846675
KQ76_RS07375		phage major capsid protein	0.844868
KQ76_RS06755		ATP-binding cassette domain-containing protein	0.808771
KQ76_RS02580	rplK	50S ribosomal protein L11	0.801481
KQ76_RS07325		phage tail family protein	0.777179
KQ76_RS03210	graS	histidine kinase GraS/ApsS	0.770472
KQ76_RS09175		DUF4352 domain-containing protein	0.76621
KQ76_RS02590	rplJ	50S ribosomal protein L10	0.762473
KQ76_RS05605	ftsA	cell division protein FtsA	0.758464
KQ76_RS05745		hypothetical protein	0.713196
KQ76_RS03215	vraF	ABC transporter ATP-binding protein VraF	0.703157
KQ76_RS07610		site-specific integrase	0.701294
KQ76_RS10640	cls	cardiolipin synthase	0.699256
KQ76_RS08860		hypothetical protein	0.676057

KQ76_RS07700		AraC family transcriptional regulator	0.638272
KQ76_RS12190		YbgA family protein	0.624883
KQ76_RS01145		hypothetical protein	0.623133
KQ76_RS05305	isdA	LPXTG-anchored heme-scavenging protein IsdA	0.563751
KQ76_RS06195	glpK	glycerol kinase GlpK	0.556582
KQ76_RS05820	fapR	transcription factor FapR	0.546234
KQ76_RS10645		HD domain-containing protein	0.526614
KQ76_RS06100		SDR family NAD(P)-dependent oxidoreductase	0.500085
KQ76_RS08715	ald	alanine dehydrogenase	0.495937
KQ76_RS11320	rpsI	30S ribosomal protein S9	0.494267
KQ76_RS07280		hypothetical protein	0.477356
KQ76_RS04415	fabF	beta-ketoacyl-ACP synthase II	0.471705
KQ76_RS07460		DUF1024 family protein	0.469275
KQ76_RS05770	rlmN	23S rRNA (adenine(2503)-C(2))-methyltransferase RlmN	0.454999
KQ76_RS02745	hxlA	3-hexulose-6-phosphate synthase	0.446456
KQ76_RS06705		ABC transporter permease	0.43506
KQ76_RS06230		aminotransferase class I/II-fold pyridoxal phosphate-dependent enzyme	0.423676
KQ76_RS12695	cntL	D-histidine (S)-2-aminobutanoyltransferase CntL	-0.48559
KQ76_RS11100		UDPGP type 1 family protein	-0.51493
KQ76_RS02485	pdxT	pyridoxal 5'-phosphate synthase glutaminase subunit PdxT	-0.51761
KQ76_RS01875		superantigen-like protein SSL1	-0.52909
KQ76_RS10360		LacI family DNA-binding transcriptional regulator	-0.55212
KQ76_RS11585	moaA	GTP 3',8-cyclase MoaA	-0.57951
KQ76_RS11590	mobA	molybdenum cofactor guanylyltransferase MobA	-0.58293
KQ76_RS09335	sei	staphylococcal enterotoxin type I	-0.62108
KQ76_RS00805		response regulator transcription factor	-0.63308
KQ76_RS01930		restriction endonuclease subunit S	-0.64915
KQ76_RS00280		DUF1648 domain-containing protein	-0.65494
KQ76_RS00645		YagU family protein	-0.67155
KQ76_RS11550		hypothetical protein	-0.67641
KQ76_RS00480		XRE family transcriptional regulator	-0.69019
KQ76_RS03890		hypothetical protein	-0.69421
KQ76_RS13325		aspartate 1-decarboxylase	-0.71437
KQ76_RS13940		DUF3147 family protein	-0.72246
KQ76_RS01530		ABC-2 transporter permease	-0.75301
KQ76_RS01275		DUF4467 domain-containing protein	-0.78786
KQ76_RS13605	manA	mannose-6-phosphate isomerase, class I	-0.81023
KQ76_RS01910		superantigen-like protein SSL9	-0.81606
KQ76_RS12425		6-carboxyhexanoate--CoA ligase	-0.84205
KQ76_RS10190		TDT family transporter	-0.84813
KQ76_RS12215		DUF3139 domain-containing protein	-0.88081
KQ76_RS01915		superantigen-like protein SSL10	-0.89397
KQ76_RS15025		transposase	-0.93004

KQ76_RS12320	adcA	zinc ABC transporter substrate-binding lipoprotein AdcA	-0.95414
KQ76_RS00795		isoprenylcysteine carboxyl methyltransferase family protein	-0.9687
KQ76_RS12285	nirD	nitrite reductase small subunit NirD	-0.98992
KQ76_RS12415	hlgB	bi-component gamma-hemolysin HlgAB/HlgCB subunit B	-1.06645
KQ76_RS13440	nrdG	anaerobic ribonucleoside-triphosphate reductase activating protein	-1.14315
KQ76_RS05525		YfcC family protein	-1.2518
KQ76_RS11300	alsS	acetolactate synthase AlsS	-1.28025

Supplementary Table 3. Unique DEGs identified in the planktonic vs unmodified Ti comparison. GeneIDs are specific to *S. aureus* ATCC25923 reference genome, as listed on the NCBI database. Gene symbols are provided where available and were acquired from the annotated reference genome. DEGs are sorted by log fold-change, where a positive value corresponds to an upregulation on unmodified Ti compared to planktonic.

GeneID	Symbol	Product Description	Log FC
KQ76_RS09980	scpA	cysteine protease staphopain A	5.113875
KQ76_RS00880		acyl CoA:acetate/3-ketoacid CoA transferase	3.502256
KQ76_RS06420		homoserine dehydrogenase	3.212019
KQ76_RS06825		5-bromo-4-chloroindolyl phosphate hydrolysis family protein	2.758667
KQ76_RS02615		ribosomal L7Ae/L30e/S12e/Gadd45 family protein	2.517145
KQ76_RS07285	lukF-PV	Panton-Valentine bi-component leukocidin subunit F	2.402489
KQ76_RS07950		superoxide dismutase	2.351649
KQ76_RS08670	accD	acetyl-CoA carboxylase, carboxyltransferase subunit beta	2.334499
KQ76_RS05960	hslU	ATP-dependent protease ATPase subunit HslU	2.320309
KQ76_RS02620	rpsL	30S ribosomal protein S12	2.314879
KQ76_RS01090	rbsD	D-ribose pyranase	2.256099
KQ76_RS06935	msrA	peptide-methionine (S)-S-oxide reductase MsrA	2.156539
KQ76_RS02625	rpsG	30S ribosomal protein S7	2.103091
KQ76_RS13615		amidase domain-containing protein	2.10054
KQ76_RS01150		CHAP domain-containing protein	2.023817
KQ76_RS13980	rpmH	50S ribosomal protein L34	1.851371
KQ76_RS07810		Asp23/Gls24 family envelope stress response protein	1.776191
KQ76_RS10655	yidC	membrane protein insertase YidC	1.68573
KQ76_RS10910	deoD	purine-nucleoside phosphorylase	1.684355
KQ76_RS12810		Hypothetical protein	1.671551
KQ76_RS13190	isaA	lytic transglycosylase IsaA	1.65213
KQ76_RS08590		replication initiation and membrane attachment family protein	1.614407
KQ76_RS01805		Hypothetical protein	1.605025
KQ76_RS04510	mecA	adaptor protein MecA	1.584948

KQ76_RS03555		5'-3'-deoxyribonucleotidase	1.57959
KQ76_RS11490	rpsJ	30S ribosomal protein S10	1.451716
KQ76_RS08950	dat	D-amino-acid transaminase	1.375269
KQ76_RS01020	tarS	poly(ribitol-phosphate) beta-N-acetylglucosaminyltransferase	1.368601
KQ76_RS12220		MarR family transcriptional regulator	1.318169
KQ76_RS07655	xerD	site-specific tyrosine recombinase XerD	1.29262
KQ76_RS11715	sarR	HTH-type transcriptional regulator SarR	1.232636
KQ76_RS06045	infB	translation initiation factor IF-2	1.213806
KQ76_RS08765		GAF domain-containing protein	1.20107
KQ76_RS01655		primase alpha helix C-terminal domain-containing protein	1.198253
KQ76_RS08595	nrdR	transcriptional regulator NrdR	1.189821
KQ76_RS07130		Hypothetical protein	1.185884
KQ76_RS01180	essC	type VII secretion protein EssC	1.185822
KQ76_RS13245		amidohydrolase family protein	1.150507
KQ76_RS01125	lytM	glycine-glycine endopeptidase LytM	1.150078
KQ76_RS03675		undecaprenyl/decaprenyl-phosphate alpha-N-acetylglucosaminyl 1-phosphate transferase	1.132165
KQ76_RS10440	ilvC	ketol-acid reductoisomerase	1.032815
KQ76_RS01005		D-ribitol-5-phosphate cytidylyltransferase	1.003806
KQ76_RS03120		ABC transporter ATP-binding protein/permease	0.994827
KQ76_RS05595	murD	UDP-N-acetylmuramoyl-L-alanine--D-glutamate ligase	0.984699
KQ76_RS08970		polysaccharide biosynthesis protein	0.984495
KQ76_RS10955		Hypothetical protein	0.974307
KQ76_RS05175		Nramp family divalent metal transporter	0.972957
KQ76_RS08055		Hypothetical protein	0.94747
KQ76_RS07015		5'-3' exonuclease	0.946654
KQ76_RS06010	rseP	RIP metalloprotease RseP	0.910551
KQ76_RS10025		SLC13 family permease	0.888286
KQ76_RS10290		site-specific integrase	0.841203
KQ76_RS13910		IS30 family transposase	0.835257
KQ76_RS11360	rpsK	30S ribosomal protein S11	0.832859
KQ76_RS01345		ROK family protein	0.781308
KQ76_RS07990	rpoD	RNA polymerase sigma factor RpoD	0.752628
KQ76_RS04160	dltB	PG:teichoic acid D-alanyltransferase DltB	0.747144
KQ76_RS11350	rplQ	50S ribosomal protein L17	0.728976
KQ76_RS03330		Hypothetical protein	0.715446
KQ76_RS09045	ribD	bifunctional diaminohydroxyphosphoribosylaminopyrimidine deaminase/5-amino-6-(5-phosphoribosylamino)uracil reductase RibD	0.701207
KQ76_RS07580		DUF739 family protein	0.659698
KQ76_RS11820		YafY family transcriptional regulator	0.653243
KQ76_RS11355		DNA-directed RNA polymerase subunit alpha	0.650514
KQ76_RS07135	aroA	3-phosphoshikimate 1-carboxyvinyltransferase	0.647774

KQ76_RS06595	mprF	bifunctional lysylphosphatidylglycerol flippase/synthetase MprF	0.635113
KQ76_RS06790		alanine racemase	0.628968
KQ76_RS07505		Hypothetical protein	0.621101
KQ76_RS06565	parC	DNA topoisomerase IV subunit A	0.586945
KQ76_RS09800		acyl-CoA thioesterase	0.542792
KQ76_RS04905		osmotic stress response protein	0.538974
KQ76_RS04170	dltD	D-alanyl-lipoteichoic acid biosynthesis protein DltD	0.537298
KQ76_RS03230		inorganic phosphate transporter	0.512248
KQ76_RS09455	yhaM	3'-5' exoribonuclease YhaM	0.508467
KQ76_RS13330	panC	pantoate--beta-alanine ligase	-0.50484
KQ76_RS11725		PH domain-containing protein	-0.53643
KQ76_RS02530		PIN/TRAM domain-containing protein	-0.54933
KQ76_RS02255	rsmA	16S rRNA (adenine(1518)-N(6)/adenine(1519)-N(6))-dimethyltransferase RsmA	-0.55135
KQ76_RS01225		TIGR01741 family protein	-0.61338
KQ76_RS00120		MBL fold metallo-hydrolase	-0.62953
KQ76_RS10825		aldehyde dehydrogenase family protein	-0.66492
KQ76_RS11955		galactose mutarotase	-0.69782
KQ76_RS12160		DUF2871 domain-containing protein	-0.71906
KQ76_RS01405		lipoate--protein ligase	-0.76489
KQ76_RS00850		Hypothetical protein	-0.78275
KQ76_RS04715		Hypothetical protein	-1.08626
KQ76_RS08305		SAS049 family protein	-1.17631
KQ76_RS11030		tRNA-Gln	-1.18244

Supplementary Table 4. Unique DEGs identified in the planktonic vs nanospiked Ti comparison. GeneIDs are specific to *S. aureus* ATCC25923 reference genome, as listed on NCBI database. Gene symbols are provided where available and were acquired from the annotated reference genome. DEGs are sorted by log fold-change, where a positive value corresponds to an upregulation on nanospiked Ti compared to planktonic.

GeneID	Symbol	Product Description	Log FC
KQ76_RS05980		Hypothetical protein	2.555589
KQ76_RS07475		DUF3113 family protein	2.449168
KQ76_RS07595		Hypothetical protein	2.218871
KQ76_RS00290	spa	staphylococcal protein A	2.167831
KQ76_RS09240		DUF1433 domain-containing protein	2.141849
KQ76_RS08560	rpmI	50S ribosomal protein L35	2.098789
KQ76_RS15110		Hypothetical protein	1.877204
KQ76_RS13860	cna	collagen adhesin Cna	1.854876
KQ76_RS04990	purF	amidophosphoribosyltransferase	1.558328
KQ76_RS04960	purE	5-(carboxyamino)imidazole ribonucleotide mutase	1.523435
KQ76_RS07480		DUF1064 domain-containing protein	1.495138
KQ76_RS08435		Hypothetical protein	1.40529

KQ76_RS07675		Hypothetical protein	1.398757
KQ76_RS02865		Rrf2 family transcriptional regulator	1.364921
KQ76_RS05160		spermidine/putrescine ABC transporter substrate-binding protein	1.24288
KQ76_RS07495		ATP-binding protein	1.096448
KQ76_RS11910	hutI	imidazolonepropionase	0.911249
KQ76_RS13120	pruA	L-glutamate gamma-semialdehyde dehydrogenase	0.851765
KQ76_RS07500		conserved phage C-terminal domain-containing protein	0.850492
KQ76_RS07575		transcriptional regulator	0.812446
KQ76_RS05410	sdhB	succinate dehydrogenase iron-sulfur subunit	0.804421
KQ76_RS07445		DUF1381 domain-containing protein	0.736383
KQ76_RS08220		acetyl-CoA carboxylase biotin carboxyl carrier protein subunit	0.68408
KQ76_RS01605	ssb	single-stranded DNA-binding protein	0.678081
KQ76_RS03630		UDP-N-acetylmuramate dehydrogenase	0.662163
KQ76_RS06900		GNAT family N-acetyltransferase	0.653498
KQ76_RS07555		Hypothetical protein	0.653268
KQ76_RS06950		thymidylate synthase	0.60972
KQ76_RS03320		TIGR00730 family Rossman fold protein	0.559462
KQ76_RS07245		ferredoxin	0.549694
KQ76_RS13075		hydroxymethylglutaryl-CoA synthase	0.519838
KQ76_RS07465		phi PVL orf 51-like protein	0.513307
KQ76_RS07345		Ig-like domain-containing protein	0.469806
KQ76_RS09720		glutamate-1-semialdehyde 2,1-aminomutase	0.462793
KQ76_RS12380		2,3-diphosphoglycerate-dependent phosphoglycerate mutase	-0.37241
KQ76_RS02350	hpt	hypoxanthine phosphoribosyltransferase	-0.3979
KQ76_RS05935	dprA	DNA-processing protein DprA	-0.42096
KQ76_RS08455		A24 family peptidase	-0.45714
KQ76_RS00205		TetR/AcrR family transcriptional regulator	-0.47698
KQ76_RS02935		DUF1934 domain-containing protein	-0.47713
KQ76_RS05535		TDT family transporter	-0.4862
KQ76_RS02880		DUF443 domain-containing protein	-0.49468
KQ76_RS13340		oxidoreductase	-0.51852
KQ76_RS11085	sepA	multidrug efflux transporter SepA	-0.51959
KQ76_RS02895		DUF443 family protein	-0.52915
KQ76_RS01725		Abi family protein	-0.55537
KQ76_RS13815		energy-coupling factor transporter transmembrane protein EcfT	-0.55653
KQ76_RS02280	spoVG	septation regulator SpoVG	-0.56215
KQ76_RS00150		Hypothetical protein	-0.56732
KQ76_RS11095		hemolysin III family protein	-0.57857
KQ76_RS12325		Txe/YoeB family addiction module toxin	-0.59744
KQ76_RS09215		DUF1433 domain-containing protein	-0.62965
KQ76_RS01220		Hypothetical protein	-0.64028
KQ76_RS12790		tandem-type lipoprotein	-0.67805
KQ76_RS15240		Hypothetical protein	-0.70879

KQ76_RS04390		Hypothetical protein	-0.73118
KQ76_RS14290		Hypothetical protein	-0.74717
KQ76_RS01615		type II toxin-antitoxin system PemK/MazF family toxin	-0.74989
KQ76_RS00490	capA	capsular polysaccharide type 5/8 biosynthesis protein CapA	-0.75664
KQ76_RS09325	sen	staphylococcal enterotoxin type N	-0.79941
KQ76_RS12260	narI	respiratory nitrate reductase subunit gamma	-0.8118
KQ76_RS00710		Hypothetical protein	-0.8359
KQ76_RS03945		Hypothetical protein	-0.85758
KQ76_RS01935		superantigen-like protein SSL11	-0.8598
KQ76_RS11605	mobB	molybdopterin-guanine dinucleotide biosynthesis protein B	-0.87355
KQ76_RS04385		L-threonylcarbamoyladenylate synthase	-0.89221
KQ76_RS02695		NAD(P)H-dependent oxidoreductase	-0.89359
KQ76_RS01520		DUF3169 family protein	-0.9132
KQ76_RS12890		LysE family transporter	-0.91346
KQ76_RS09205		DUF1433 domain-containing protein	-0.92525
KQ76_RS08885		Hypothetical protein	-0.96256
KQ76_RS15225		Hypothetical protein	-0.97658
KQ76_RS12420		QueT transporter family protein	-0.97958
KQ76_RS00170	mcrC	5-methylcytosine-specific restriction endonuclease system specificity protein McrC	-1.05957
KQ76_RS15065		Hypothetical protein	-1.15972
KQ76_RS10010		nitric oxide synthase oxygenase	-1.72181
KQ76_RS00920		L-lactate dehydrogenase	-1.80673
KQ76_RS13555	arcD	arginine-ornithine antiporter	-3.74501

Table S5 lists a summary of the spectral ranges of relevant chemical bonding signatures associated with the key biochemical components. In brief, lipids are associated with ν(C-H) stretching vibrations of methyl ($-CH_3$) and methylene ($-CH_2$) groups dominated in the fatty acid structure. Proteins, on the other hand, can be identified by amide I and II bands that represent ν(C=O) stretches and δ(N-H) deformation (in primary amides) or a combination of δ(N-H) bending and ν(C-N) stretching vibrations (in secondary amides), respectively. Polysaccharides are associated with ν(C-O) stretching modes coupled with δ(C–O) of C–OH groups in 1° and 2° alcohols of carbohydrates. Furthermore, lysyl-phosphatidylglycerol (LPG) can be assigned based on ν(C-N) stretching and δ(N-H) bending vibrations of its additional amine groups, whilst D-alanylated teichoic acids leads to ν(C-O-C) vibration associated with esters.

Supplementary Table 5. Summary of relevant *S. aureus* cell components and products, along with their chemical bond markers and associated spectral regions. Peaks identified in Fig. 4C are listed here as candidates that fit into the relevant spectral regions.

Representative biochemical components	Vibrational modes	Expected spectral region (cm^{-1})	Observed peaks (cm^{-1})
Lipids (general)	ν(C-H) stretch	3000-2840 [5]	2963, 2923, 2855
Proteins (general)	ν(C=O) stretch (amide I)	1700-1600 [5]	1659, 1643, 1633,
	δ(N-H) deformation and ν(C-N) (amide II)	1600-1450 [5]	1550, 1543, 1513, 1451
Polysaccharides (general)	ν(C-O) stretch and δ(C–O) deformation (1° and 2° alcohols)	1150-1000 [6]	1120, 1082
Lysyl-phosphatidylglycerol	ν(C-N) stretch (1° amine)	1250-1020 [7]	1167, 1120
	δ(N-H) bend (amines)	1650-1580 [7]	1643, 1633,
D-alanylated teichoic acid	ν_s(C-O-C) stretch (ester)	1210-1163 [7]	1167

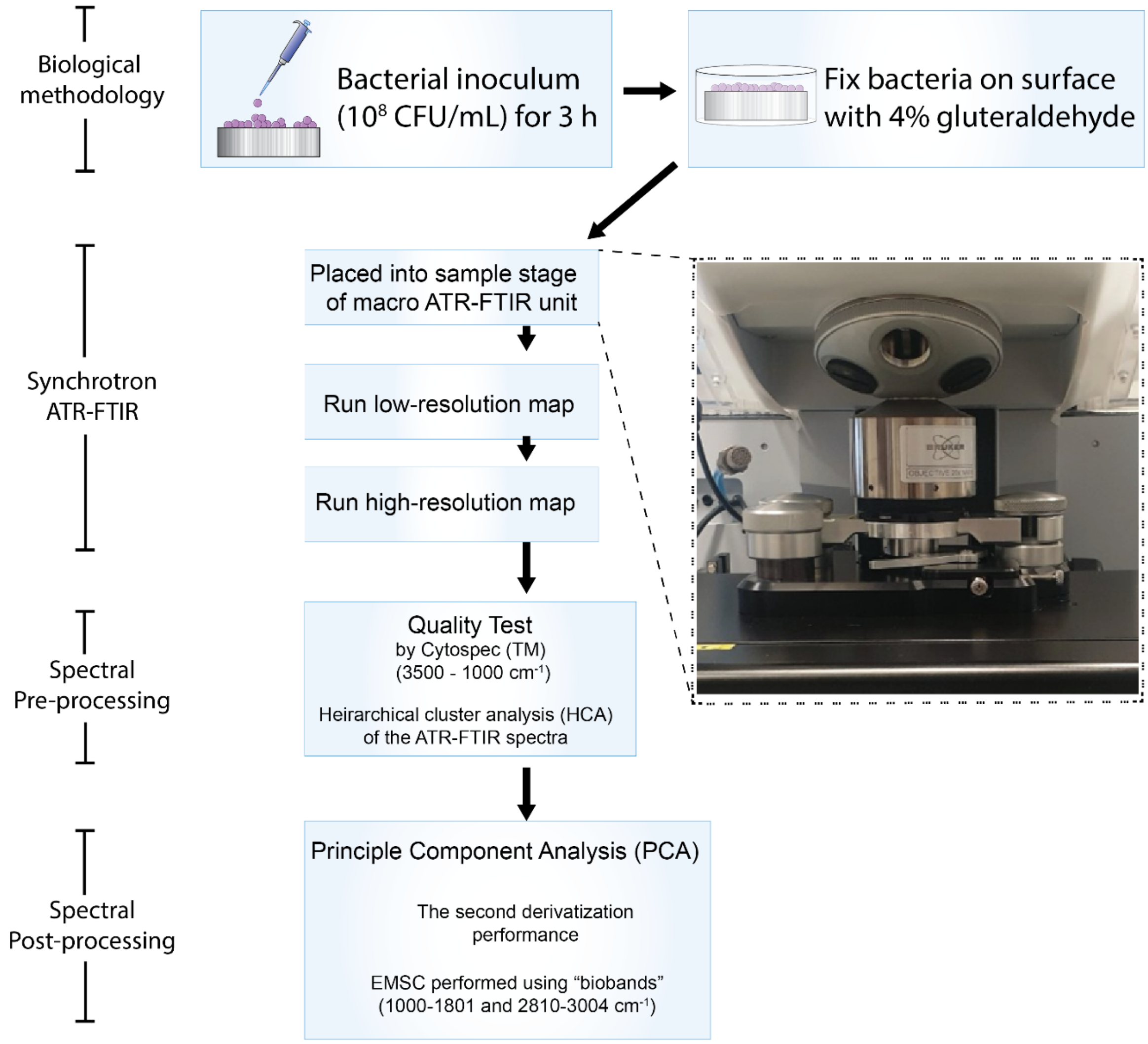

Supplementary Figure 3. The flow of analysis used to generate and process ATR-FTIR data

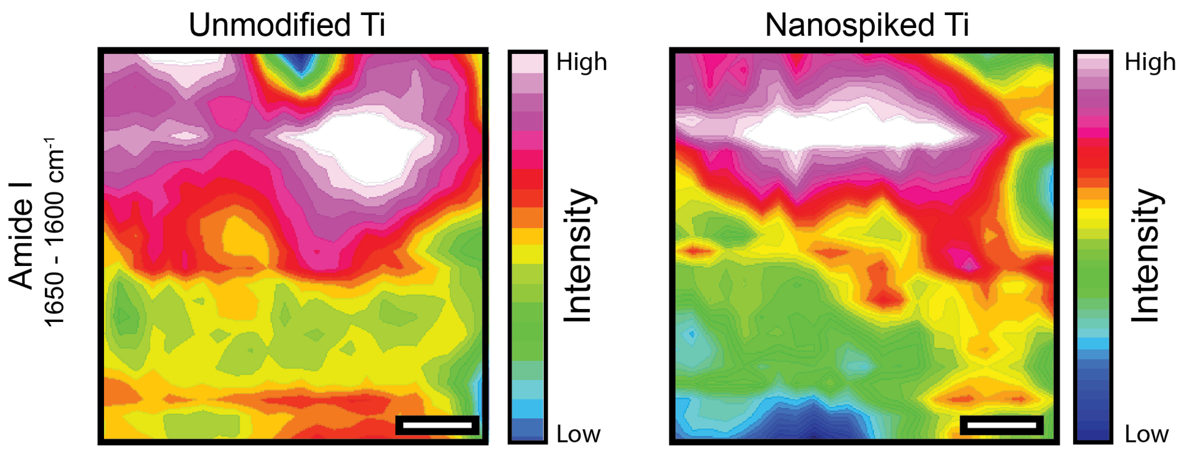

Supplementary Figure 4. Heatmaps of the Amide I region on umodified and nanospiked Ti

The activity of sub-MIC vancomycin

We investigated whether the synergy between nanospikes and vancomycin would persist if the vancomycin dosage was reduced to half its MIC value (Fig. S3). On the unmodified surface, the attachment of *S. aureus* for 3 h resulted in 80% post-treatment viability. With 6 h attachment, the post-treatment viability was approximately 95%. Contrasting this, attachment to the nanospiked surface was associated with a decrease in post-treatment viability. With a 6 h attachment, *S. aureus* had a viability of <1%.

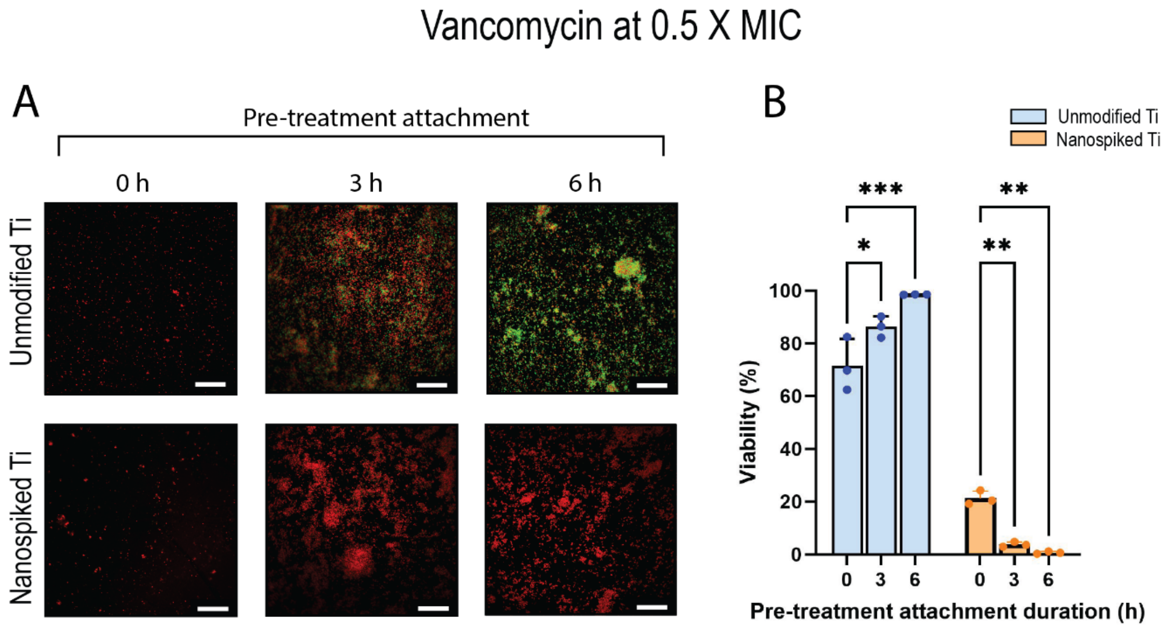

Supplementary Figure 5. Activity of sub-MIC dose vancomycin against *S. aureus* attached to the nanospiked surfaces for the increasing duration. A) The Live/Dead fluorescence micrographs obtained

following attachment and subsequent vancomycin treatment. B) The mean post-treatment viability of *S. aureus* quantified from fluorescence micrographs. Scale bars represent 30 µm. * P < 0.05, ** P < 0.01, *** P < 0.001 mean ± SD

Minimum inhibitory concentration of vancomycin

We determined the minimum inhibitory concentration of vancomycin against planktonic cells, following the standards outlined by the Clinical and Laboratory Standards Institute.[8] Vancomycin had an MIC of 1 µg/mL against *S. aureus* ATCC 25923 (Fig. S6). This value is consistent with the commonly cited breakpoints for drug sensitivity.

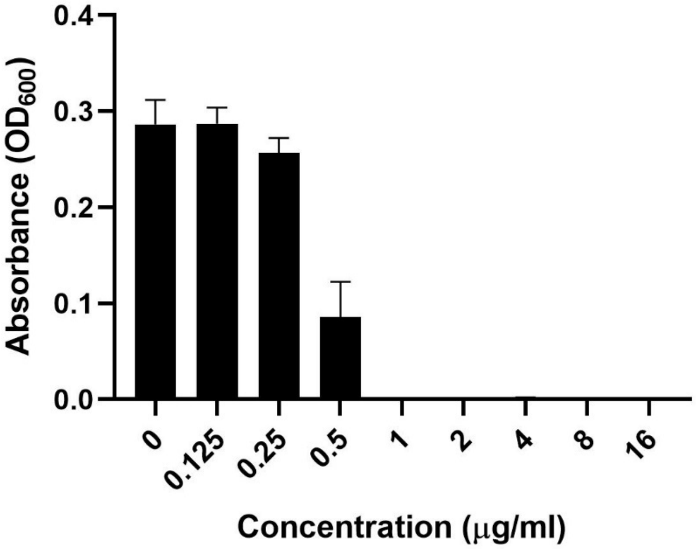

Supplementary Figure 6. Minimum inhibitory concentration of vancomycin against *S. aureus* ATCC25923

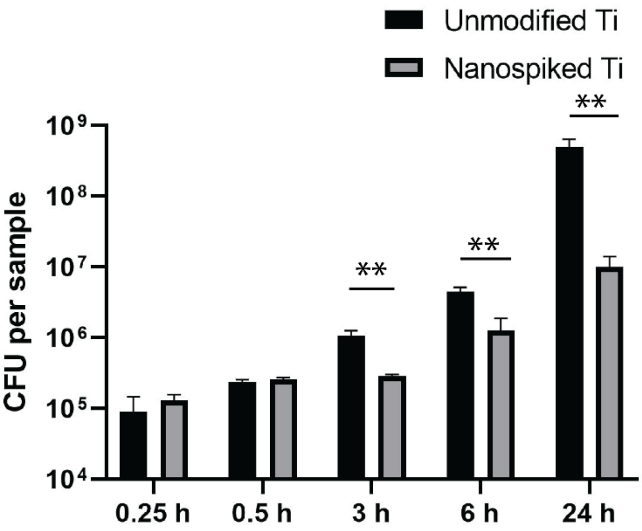

Supplementary Figure 7. Colony enumeration over 24 h on Unmodified Ti and Nanospiked Ti. n = 3 ± SD, ** $P < 0.01$

Methods

Fabrication of Nanospiked Ti surface

Ti6Al4V coupons of 10mm diameter and 3 mm height were obtained from Hamagawa Industrial SDN BHD (Kedah, Malaysia). The coupons were treated by alkaline hydrothermal processing using 1 M KOH and heated at 150 °C for 5 h in a sealed steel vessel. Samples were then rinsed in ultrapure water and annealed for 5 h.

Material characterization of Nanospiked Ti surface

Topographical dimensions of titanium nanospikes were measured using high magnification SEM images obtained on a Zeiss Merlin FEG-SEM (Jena, Germany), with a 45° stage tilt. SEM micrographs were imported into ImageJ v1.53 (NIH, USA) for measurements. EDS spectra were obtained with an x-ray spectrometer (Aztec v3.1, Oxford Instruments, MA, USA) at 15 kV.

Wettability

Atomic Force Microscopy (AFM) of a 5 x 5 µm scan area was acquired in the air using a JPK NanoWizard III with instrument-specific software v5. An NT-MDT NSG03 silicon nitride cantilever with a conical tip rated by the manufacturer at a radius < 10 nm and a half side angle of 18° was used to perform AM tapping mode on an annealed Ti6Al4V polished control surface and a hydrothermally etched KOH nanostructure surface. Non-contact calibration of the cantilever derived a normal spring constant of 1.7 N/m. Scanning parameters were set to a scan rate of 0.7 Hz at a Set Point of 27.8 nm and a drive amplitude of 0.4 Volts. Roughness values were calculated through Gwyddion data analysis software v2.54. The Ti6Al4V control sample measured RMS at 15 nm and Ra at 12 nm. The TI6Al4V KOH nanostructure surface roughness measured RMS at 87.6 nm and Ra at 70.6 nm. Tip convolution resulting from the cantilever tip side angle and scan velocity reduces the measured surface roughness values, especially towards surface features with comparable magnitude to the tip radius.

Minimum inhibitory concentration of vancomycin

Minimum inhibitory concentration of vancomycin against *S. aureus* ATCC25923 was determined following the standards set by the Clinical and Laboratory Standards Institute (CLSI).[8] The protocol was adapted to use TSB in place of cation-adjusted Mueller-Hinton Broth. Briefly, *S. aureus* was incubated in TSB with a seeding density of 5×10^5 cfu/mL and treated with two-fold serially diluted antibiotics between the concentrations of 0.125 and 16 µg/mL. The plates were then incubated at 37°C for 24 h, and the final cell density was measured by OD_{600} in a Synergy HTX Multi-Mode microplate reader (BioTek Instruments, Winooski, Vermont, USA).

Colony enumeration

Unmodified and nanospiked Ti samples were placed in the wells of a sterile 24-well plate, and immersed in a suspension of *S. aureus* ATCC2593 at a cell density of 10^8 CFU/ml. At timepoints of 0.25, 0.5, 3, 6 and 24 h, samples were removed from the cell suspension and gently rinsed in sterile PBS to remove non-adherent cells. Cells were then detached by sonication (2 mins) and vortex (30 seconds) in 1 mL of sterile PBS. The resulting suspensions were serially diluted and dropped in triplicate on TSB agar plates in 10 µL aliquots. Colonies were counted the following day.

References

1. Bright R, Hayles A, Fernandes D, Visalakshan RM, Ninan N, Palms D, et al. In Vitro Bactericidal Efficacy of Nanostructured Ti6Al4V Surfaces is Bacterial Load Dependent. *ACS Applied Materials & Interfaces*. 2021;**13**(32):38007-17.
2. Marshall CG, Broadhead G, Leskiw BK, Wright GD. D-Ala-D-Ala ligases from glycopeptide antibiotic-producing organisms are highly homologous to the enterococcal vancomycin-resistance ligases VanA and VanB. *Proc Natl Acad Sci U S A*. 1997;**94**(12):6480-3.
3. Jia Z, O'Mara ML, Zuegg J, Cooper MA, Mark AE. Vancomycin: ligand recognition, dimerization and super-complex formation. *The FEBS Journal*. 2013;**280**(5):1294-307.
4. Po HN, Senozan NM. The Henderson-Hasselbalch Equation: Its History and Limitations. *Journal of Chemical Education*. 2001;**78**(11):1499.
5. Böcker U, Ofstad R, Wu Z, Bertram HC, Sockalingum GD, Manfait M, et al. Revealing covariance structures in fourier transform infrared and Raman microspectroscopy spectra: a study on pork muscle fiber tissue subjected to different processing parameters. *Appl Spectrosc*. 2007;**61**(10):1032-9.
6. Movasaghi Z, Rehman S, ur Rehman DI. Fourier Transform Infrared (FTIR) Spectroscopy of Biological Tissues. *Applied Spectroscopy Reviews*. 2008;**43**(2):134-79.
7. Merck. IR Spectrum Table: Sigma Aldrich; 2022 [Available from: https://www.sigmaaldrich.com/AU/en/technical-documents/technical-article/analytical-chemistry/photometry-and-reflectometry/ir-spectrum-table.
8. CLSI. Performance Standards for Antimicrobial Susceptibility Testing. 30th ed: Clinical and Laboratory Standards Institute; 2020.
